# An activity-modulated transport route across myelin (TRAM) for motor-driven organelle transfer in oligodendrocytes

**DOI:** 10.1038/s41593-026-02383-0

**Published:** 2026-07-31

**Authors:** Katie J. Chapple, Tabitha R. F. Green, Kristen H. Schuster, Sarah Wirth, Yi-Hsin Chen, Ulrike C. Gerwig, Marie Louise Aicher, Yeonsu Kim, Lina Komarek, Angus Brown, Colin L. Crawford, Rebecca Sherrard Smith, Jeffrey Y. Lee, Lauren E. Black, Luis Pardo, Rebecca E. McHugh, Celia M. Kassmann, Wiebke Möbius, Hauke B. Werner, Ilan Davis, Matthias Kneussel, Ethan G. Hughes, Euan R. Brown, Sandra Goebbels, Klaus-Armin Nave, Julia M. Edgar

**Affiliations:** 1 School of Infection & Immunity, College of Medical Veterinary and Life Sciences, University of Glasgow, Glasgow, UK; 2 Department of Cell and Developmental Biology, University of Colorado, School of Medicine, Aurora, CO USA; 3 Dept of Neurogenetics, Max Planck Institute for Multidisciplinary Sciences, City Campus, Göttingen, Germany; 4 School of Life Sciences, University of Nottingham, Nottingham, UK; 5 School of Molecular Biosciences, College of Medical Veterinary and Life Sciences, University of Glasgow, Glasgow, UK; 6 Electron Microscopy-City Campus, Max Planck Institute for Multidisciplinary Sciences, Göttingen, Germany; 7 Faculty of Biology and Psychology, University of Göttingen, Göttingen, Germany; 8 Department of Molecular Neurogenetics, Center for Molecular Neurobiology, ZMNH, University Medical Center Hamburg-Eppendorf, Hamburg, Germany; 9 School of Engineering and Physical Sciences, Institute of Biological Chemistry, Biophysics and Bioengineering, Heriot Watt University, Edinburgh, UK; 10 Department of Neuropathology, Charité Universitätsmedizin, Berlin, Germany

**Keywords:** Multiple sclerosis, Microtubules, Neuroscience

## Abstract

Myelin sheaths comprise compacted layers of membrane wrapped around axons. Each sheath, if ‘unwrapped’, has a cytoplasm-filled space at its perimeter. Transmission electron microscopy reveals that this space contains microtubules and organelles; however, whether these are developmental remnants or serve a specific function remains unknown. Live imaging of myelinating oligodendrocytes in mice showed microtubule-dependent organelle transport within myelin sheath cytoplasmic spaces. Further, movement of myelin-located peroxisomes was modulated by neuronal electrical activity, in vitro and in vivo. Loss of oligodendroglial KIF21B and/or CNP in vivo led to the apparent stasis of myelin organelles and secondary axon pathology. We, thus, propose the acronym TRAM (transport route across myelin), defining the continuous cytoplasmic network in oligodendrocytes and myelin. This system, comprising the inner and outer tongues and paranodal loops, spans compact myelin and enables transport of metabolites and organelles between the oligodendrocyte soma and the periaxonal space.

## Abstract (web summary)

Axons require intracellular transport of cargoes between the soma and the terminus. The authors show a related logistical challenge exists at smaller scale with respect to the interaction of process-bearing oligodendrocytes and the axons they myelinate.

## Main

**Fig. 1:**
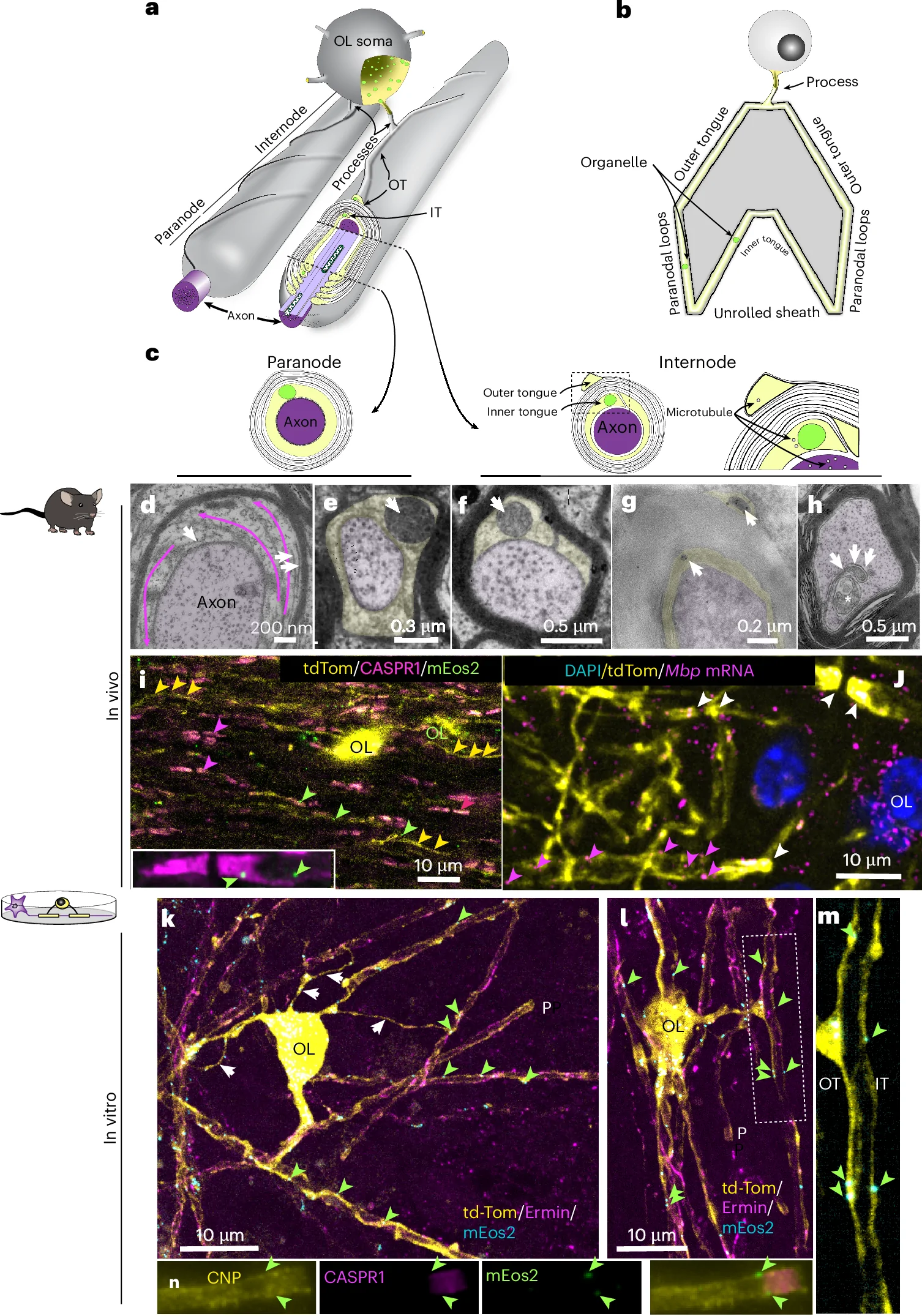
Microtubules and membranous organelles in myelin’s cytoplasmic spaces. **a**–**c**, Schematic of an oligodendrocyte soma (OL) and myelin sheaths (**a**). Myelin’s cytoplasmic spaces (yellow) include inner tongues (IT) and outer tongues (OT) that entwine the myelin sheath on its inner and outer surfaces, respectively, and the axon-tethered paranodal loops. Dashed lines depict approximately where the corresponding TEM cross-sections are located. Our working model of the unrolled sheath^13^ (**b**). The IT, OT and paranodal loops form a continuous space at the perimeter of the sheath that are linked with the OL via the cell process. Cross-sections through the paranode and internode, corresponding to the TEMs in **d**–**h** (**c**). The inset shows that these cytoplasm-filled spaces contain microtubules, and a small number of organelles (green). **d**–**h**, TEMs of myelinated axon cross-sections including paranodal loops (**d**,**e**), IT (**f**), IT and OT (**g**) and invagination of myelin membrane into the axon (**h**). White arrows indicate microtubules in paranodal loops (**d**), which encircle the axon (magenta arrows). In **e**, a dense organelle resembling a lysosome resides in a paranodal loop. Multivesicular body occupies the IT (**f**). Immuno-electron microscopy with anti-catalase (10-nm gold particles) and PMP70 (15-nm gold particles), labeling peroxisomes in the IT and (presumed) OT (**g**). Invagination of glial cell membrane (arrows) surrounding an abnormal-appearing axonal organelle (asterisk), shown previously in the peripheral nervous system (PNS)^26^ (**h**). 0.82 ± 0.45 of myelinated axons contained dense organelles in ITs. 18.02 ±12.5% of paranodes contained dense organelles. >2,300 myelinated axons examined (*n* = 4 optic nerves, 7–12-month-old mice). **i**, Maximum intensity projection (MIP) from a confocal *z*-stack of a longitudinal section of adult mouse optic nerve. The OL and myelin (yellow arrowheads) are labeled with tdTomato (tdTom) (pseudocolored yellow). Paranodes are identified with anti-CASPR1 (magenta; magenta arrowheads), located on the axon. OL peroxisomes are labeled with mEos2-PTS1 (green; green arrowheads). Peroxisomes are observed in the somata (in left OL, they are obscured by tdTomato) and myelin sheaths, including paranodes. Higher magnification of individual channels Extended Data Fig. 1l. Inset shows two CASPR1 stained paranodes separated by a node of Ranvier. By inference, the two peroxisomes are in paranodal and internodal myelin, respectively. **j**, MIP from three confocal *z*-steps of the cerebral cortex of a 1-year-old mouse. Single-molecule fluorescent in situ hybridization (smFISH) was used to visualize *Mbp* mRNA (magenta). tdTomato (pseudocolored yellow) fills the IT and OT and paranodal loops of myelin (the last indicated by white arrowheads). *Mbp* mRNA is observed around the right-most nucleus (4,6-diamidino-2-phenylindole (DAPI); blue), in the IT and OT (magenta arrowheads on sheath on bottom left) and in the paranodes (white arrowheads). Individual channels shown in Extended Data Fig. 1n. **k**, MIP of myelinating OL in vitro labeled with tdTomato (pseudocolored yellow) and anti-Ermin (magenta), a cytoskeletal molecule that first appears during late myelination stages^28^. mEos2-PTS1-labeled peroxisomes (pseudocolored cyan) are observed in oligodendrocyte processes (white arrows) and myelin sheaths (green arrows). **l**, MIP of second OL in vitro showing peroxisomes (green arrows) in myelin sheaths. In sheath delineated by a dotted line, note a triangular shaped thickening at the junction between the oligodendrocyte process and OT. Extended Data Fig. 1 shows individual channels, and also Supplementary Videos 1 and 2. **m**, High-magnification view of myelin sheath delineated by dotted line in **l**. Peroxisomes are in both OT and IT. OT can be identified by its relationship to the oligodendrocyte process (**a**,**c**). By inference, the IT is opposite. **n**, Paranodal region from an oligodendrocyte in vitro labeled with anti-CNP (myelin, yellow) and anti-CASPR1 (on the axon, magenta). Two mEos2-PTS1-labeled peroxisomes are visible (green arrows); by inference, the bottom one is located just inside the paranodal region, marked by CASPR1, and top one at the juxtaparanode, between the internode and paranode.

In the nervous system, the unusual size and architecture of long axons require intracellular transport for moving cargoes between the somata and axonal terminals^1^. Here we show that a related logistical challenge exists at a much smaller scale with respect to the vital interaction of process-bearing oligodendrocytes and the axons they myelinate. Compacted myelin decreases the transverse capacitance and increases the electrical resistance across the myelinated fiber; however, myelin also separates the oligodendrocyte soma from the internodal axon–glial junction (Fig. 1a), a 20-nm wide gap thought to mediate the bicellular exchange of metabolites through monocarboxylate transporters^2,3^.

In the central nervous system (CNS), most myelin sheaths derive from a single lamellipod that extends from the oligodendrocyte soma; although exceptions occur on branched axons^4^. Growth of the prospective sheath involves actin remodeling at the lamellipod’s leading edge (future ‘inner tongue’), which advances around the axon, underneath accumulating membrane wraps^5–7^. Simultaneously, the lamellipod grows along the axon’s length in response to calcium signaling in the oligodendrocyte^8,9^. Membrane compaction occurs by polymerization of myelin basic protein (MBP) between opposing intracellular surfaces^10^, combined with tethering of opposing extracellular surfaces culminating in tightly wrapped concentric layers of membrane; however, a cytoplasm-filled space remains at the perimeter of the sheath (Fig. 1b), suggesting a direct connection to the intracellular contents of the cell soma via the cell process^11^ (Fig. 1a).

This cytoplasm-filled space forms the ‘inner and outer tongues’ at the innermost and outermost edges of the wrapped sheath and the paranodal loops at either end of the sheath (Fig. 1a–c). Other cytoplasm-filled spaces include transient openings of the compacted myelin^12^, rare Schmidt–Lanterman incisure-like openings on large fibers and other conformations, not yet defined (reviewed elsewhere^13^). The myelin sheath’s cytoplasm-filled spaces are generated and maintained, at least in part, by 2′,3′-cyclic nucleotide 3′-phosphodiesterase (CNP; encoded by *Cnp*, also known as *Cnp1*)^12^.

Myelin’s cytoplasmic spaces contain microtubules, endoplasmic reticulum, vesicle-like structures, multivesicular bodies, peroxisomes and Golgi outposts^14–17^. As all these compartments have been implicated in developmental myelination, it remains unknown whether they exist as remnants of myelinogenesis, captured in the mature sheath, or have a functional role. We previously speculated that in the adult, myelin’s cytoplasmic spaces provide a route for metabolites to reach the glial–axonal junction^18–20^. Furthermore, as active transport processes can be visualized in cytosolic spaces of flattened myelin-like sheets in oligodendrocyte monolayer cultures^21^, we additionally hypothesized that myelin’s cytoplasmic spaces might serve as a route for the movement of membranous cargo to the internodal glial–axonal junction, analogous to axonal transport to synaptic terminals.

Here we demonstrate microtubule-dependent organelle transport in established myelin sheaths and show that ablation from oligodendrocytes of kinesin 21B (KIF21B), a processive motor and modulator of microtubule dynamics^22^, leads to late onset secondary axon degeneration in vivo. This coincides with reduced levels of monocarboxylate transporter 1 (MCT1) in biochemically enriched myelin. Consistent with this, MCT1 is also diminished in CNP-deficient mice and in aged mice, both of which have structural myelin defects and axonal changes^18,23^. As MCT1 resides at the glial–axonal junction and is thought to mediate the transfer of monocarboxylates between oligodendrocytes and axons^2,3^, our data suggest that TRAM is vital for oligodendrocyte-mediated axon support.

## Results

### Organelle content in myelin’s cytoplasmic space

**Extended Data Fig. 1:**
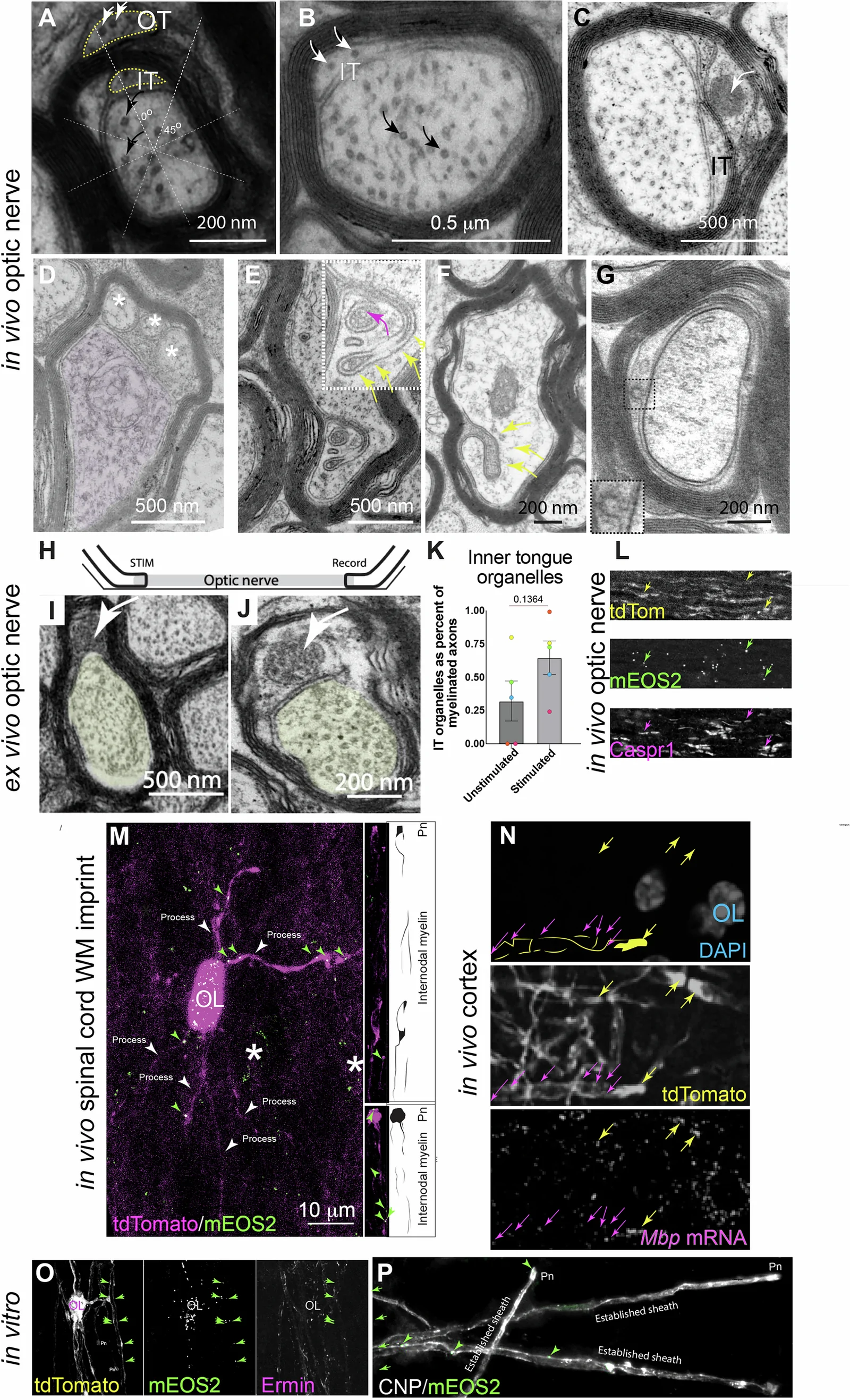
Microtubules and organelles populate the myelinic channel system. Electron micrographs of optic nerve fibers of wild-type mice. **A**. Microtubules (white arrows) in the outer tongue (OT) of myelin appear identical to those in the axon (black arrows). Here, the inner tongue (IT) and OT occur in the same octant, as delineated by dotted lines and reported previously (1). **B**. Microtubules (white arrows) in the IT and in the axon (black arrow), for comparison. **C**. A dense organelle (white arrow) is located between otherwise compacted layers of myelin. **D**. Some membrane-bound structures (asterisks) in inner tongues and paranodal loops had a pale core similar to the axoplasm, suggesting these are invading axonal sprouts (Steyer et al., 2023) **E** and **F**. Glial invaginations of the axon (yellow arrows) often (**E**), but not always (**F**) appear to envelop axonal organelles (magenta arrows). **G**. A fusion profile on the glial membrane (black square and inset) that appears to open into the periaxonal space. **H**. One of each pair of adult mouse optic nerves were held between stimulating and recording suction electrodes and stimulated at 1 Hz for one minute then 7 (blue symbols in K) or 50 Hz (pink, orange, green and yellow symbols in K) for 20 minutes. The contralateral nerve was unstimulated. **I** and **J**. Myelin organelles in the inner tongue (white arrows). **K**. On average, there was an increase in dense organelles (peroxisome-like structures as in I or multivesicular bodies as in J) in the stimulated nerve compared to the unstimulated one, although the differences were not significant. Bars represent mean $\pm \,$SD. Data were analyzed using an unpaired, two-tailed Student’s t-test. p = 0.1364. The number of fibers examined per independent optic nerve varied from 202 minimum to 414 maximum. **L**. Individual channels from Fig. 1i showing myelin peroxisomes in paranodes in the optic nerve. **M**. tdTomato-labeled oligodendrocyte from an imprint of adult murine spinal cord in which mEos2-PTS1-labeled peroxisomes (green arrowheads) can be observed in the cell body (asterisk), processes (white arrows) and in myelin sheaths (the last shown as insets on the right), where they were often located in paranodal loops (Pn). For clarity, schematics of the sheaths are shown alongside. **N**. Individual color channels from a zoomed in region of the composite image in Fig. 1j, showing *Mbp* mRNA in myelin *in vivo*, paranodes are denoted with yellow arrows. **O**. Individual color channels from composite Fig. 1l, showing a tdTomato-labeled myelinating oligodendrocyte, mEOS2-PTS1 labeled oligodendroglial peroxisomes and Ermin, *in vitro*. **P**. In another myelinating cell culture, three CNP +ve myelin sheaths terminate in paranodal loops. mEOS2-PTS1 labeled peroxisomes are present in internodal and paranodal (Pn) regions of the sheaths.

To define a putative transport infrastructure in myelin’s cytoplasmic spaces (Fig. 1a–c), we used transmission electron microscopy (TEM) of adult mouse optic nerve cross-sections. As reviewed previously^14^, some paranodal loops of myelin contained microtubules (Fig. 1d), which were also observed in inner and outer tongues (Extended Data Fig. 1a,b). Single membrane-bound dense bodies resembling lysosomes (Fig. 1e) and multivesicular bodies^10^ (Fig. 1f) were found in inner and outer tongues and paranodal loops, and very rarely, in cytosolic pockets of otherwise compact myelin (Extended Data Fig. 1c). Immuno-electron microscopy with antibodies to catalase and PMP70 labeled peroxisomes in the inner and outer tongues (Fig. 1g). Some membrane-bound structures in the inner tongues and paranodal loops had a pale core similar to the axoplasm, suggesting that these are invading axonal sprouts^24^ (Extended Data Fig. 1d). Of the 2,348 optic nerve axon cross-sections examined, 0.82 ± 0.45% (*n* = 4 optic nerves of 12-month-old mice) of inner tongue processes contained organelles. Based on (1) TEM sections being ~50 nm thick, (2) average optic nerve myelin sheath length being ~76 μm (ref. ^25^), (3) the assumption that organelles are distributed randomly, and (4) that they are spherical in shape, being ~0.3 μm in diameter (Fig. 1c,d), our data suggest that the inner tongue of each optic nerve myelin sheath contains ~two such organelles (as paranodal loops are encountered infrequently in TEM images, we were unable to determine their organelle content using this method). In addition to organelles, we observed invaginations of glial membrane into the axon (Fig. 1h and Extended Data Fig. 1e,f), similar to reports in peripheral nerves^26^, indicating these cytoplasm-filled spaces are dynamic, as shown in vitro, previously^13^. Very rarely, we observed myelin membrane-derived fusion-like profiles with the periaxonal space (Extended Data Fig. 1g), suggesting direct exchange of materials between the two compartments.

As glutamate, which is released from electrically active axons^27^, enhances the release of exosomes from oligodendrocytes in vitro^15^ we next asked whether axonal spiking influences the localization of organelles to myelin’s inner tongue, facing the axon (Fig. 1a,b) using an ex vivo optic nerve preparation to precisely control action potential firing (Extended Data Fig. 1h). One of each pair of adult mouse optic nerves was unstimulated while the contralateral nerve was stimulated at 7 or 50 Hz for 20 min. By TEM of cross-sections, the stimulated nerve had more inner tongues with organelles (mean ± s.d. of 0.65 ± 0.28% of 1,673 fiber cross-sections: ~1.7 organelles per sheath) compared to the unstimulated contralateral nerve (0.32 ± 0.34% of fibers of 1,452 fiber cross-sections; ~0.8 organelles per sheath), although with five nerve pairs (202–414 fiber cross-sections per nerve), the differences were not significant (Extended Data Fig. 1i–k). While organelle numbers could also decrease due to fusion, these data led us to speculate that organelle transport to or from the inner tongue is modulated by axonal electrical activity.

Next, we focused on peroxisomes using a transgenic mouse (*Cnp-EOS*^*SKL*^) in which oligodendroglial peroxisomes are labeled with the photoconvertible fluorescent protein mEos2, in frame with the SKL peroxisomal targeting sequence 1 (mEos2-PTS1). To demarcate myelin’s cytoplasmic spaces, we additionally labeled a proportion of oligodendrocytes with a transgenically expressed, tamoxifen-inducible fluorescent protein, tdTomato. In longitudinal sections of adult mouse optic nerves, mEos2-PTS1-labeled peroxisomes were observed in the oligodendrocyte soma; at paranodes, as indicated by axonal expression of CASPR1; and in internodal myelin (Fig. 1i and Extended Data Fig. 1l). This was confirmed in spinal cord (Extended Data Fig. 1m). *Mbp* mRNA was also observed in myelin’s cytoplasmic spaces, including the paranodal loops, in adult (1-year-old) mice (Fig. 1j and Extended Data Fig. 1n); however, in the densely packed white matter it was difficult to resolve individual sheaths, therefore, we moved to a murine spinal cord-derived ‘myelinating cell culture’, in which axons are enwrapped in compact myelin. Oligodendrocyte peroxisomes were observed in tdTomato-rendered somata, processes and sheaths (Fig. 1k), including inner and outer tongues (Fig. 1l,m, Extended Data Fig. 1 and Supplementary Videos 1 and 2) and in 48.2 ± 7.4%) of paranodes (Fig. 1n and Extended Data Fig. 1p). tdTomato often colocalized with anti-Ermin, a marker of late-developmental and mature myelin^28^ (Fig. 1k,l).

### Organelle transport in the myelin sheath is microtubule-dependent

**Fig. 2:**
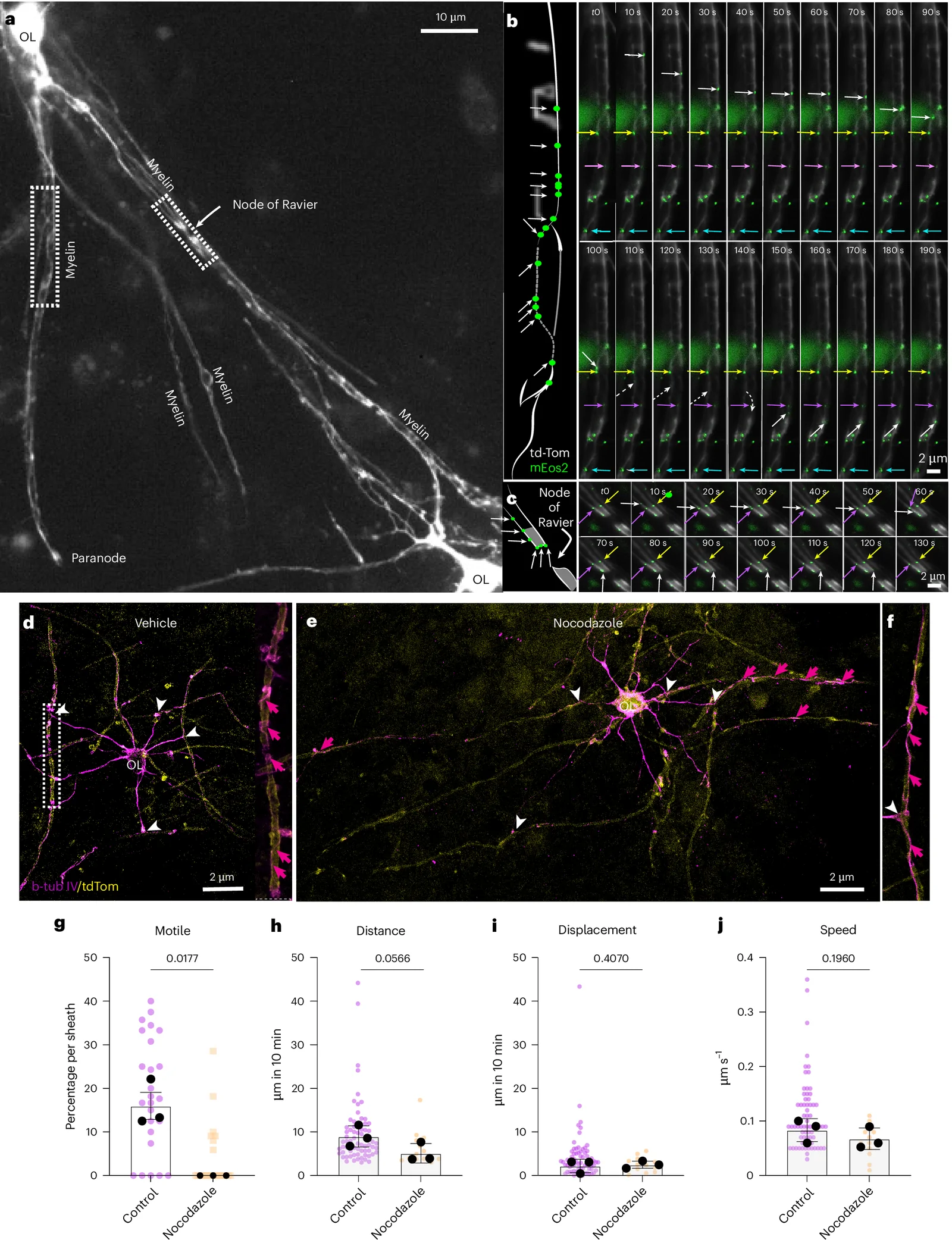
Myelin-located peroxisome transport is microtubule dependent. **a**, Fluorescence micrograph overview of two tdTomato-rendered oligodendrocytes from which the timelapse images in **b** and **c** were acquired. Under control conditions, over 43 such cells were captured by timelapse microscopy. **b**, Timelapse images showing a motile mEos2-PTS1-labeled peroxisome move through myelin’s cytoplasmic spaces toward the paranode (see overview). Schematic on the left shows the total movement of a single motile peroxisome (white arrow) during the timelapse where white lines represent cytoplasm-filled spaces within (solid lines) and outside (dashed lines) the focal plane. This peroxisome slowed or stalled at 30-, 110- and 160-s time points. At 140 s, the peroxisome moved out of the focal plane and reappeared at 150 s. Most peroxisomes (colored arrows) were stationery. **c**, Timelapse images illustrate a peroxisome (white arrow) moving down the outer tongue process (determined from relationship to the node of Ranvier) and traverse the presumptive paranodal loop nearest the node of Ranvier, before moving out of the focal plane. Other peroxisomes (colored arrows) were stationary. The left schematic illustrates the totality of movement. Paranodal regions of adjacent sheaths are separated by a node of Ranvier (**a**). **d**–**f**, β-Tubulin IV staining of tdTomato-labeled oligodendrocytes, following extraction of free tubulins (also Supplementary Video 7), treated with vehicle (**d**) or nocodazole (20 μM, 3-h incubation; **e**,**f**). White arrows indicate where processes attach to the myelin sheath and magenta arrows indicate regions of continuous β-tubulin IV staining. The myelin sheath delineated by a white rectangle in **d** is shown at higher magnification in the inset (individual channels are shown in Extended Data Fig. 2). The sheath in **f** is from a different cell from that in **e** (individual channels in Extended Data Fig. 2). **g**, Nocodazole (20 μM, 3-h incubation) significantly reduced the percentage of motile myelin peroxisomes during 10 min of live imaging. Colored symbols represent individual sheaths (control, *n* = 26, nocodazole, *n* = 24; 1–4 sheaths per cell) black symbols represent independent experimental medians (*n* = 3); bars represent mean of experimental median values ± s.d. Unpaired, one-sided, Welch’s *t*-test to compare experimental medians. **h**,**i**, Nocodazole did not significantly reduce the median distance traversed or the median displacement of motile peroxisomes, during 10 min of imaging. Nonetheless, the maximum distance reached in controls (purple dots) was not attained in the presence of nocodazole (orange dots). Displacement values were generally lower than distances reflecting that many peroxisomes reversed direction. Colored symbols represent individual peroxisomes (control, *n* = 68 and nocodazole, *n* = 13), black symbols represent independent experimental medians (*n* = 3), bars represent mean of median ± s.d. Unpaired, one-sided, Welch’s *t*-test to compare experimental medians. **j**, Nocodazole seemed not to alter the speed of motile myelin peroxisomes. Colored symbols represent individual motile peroxisomes (control, *n* = 68; nocodazole, *n* = 13), black symbols represent independent experimental medians (*n* = 3); bars represent mean of the median values± s.d. Unpaired, one-sided, Welch’s *t*-test to compare experimental medians. Source data

**Extended Data Fig. 2:**
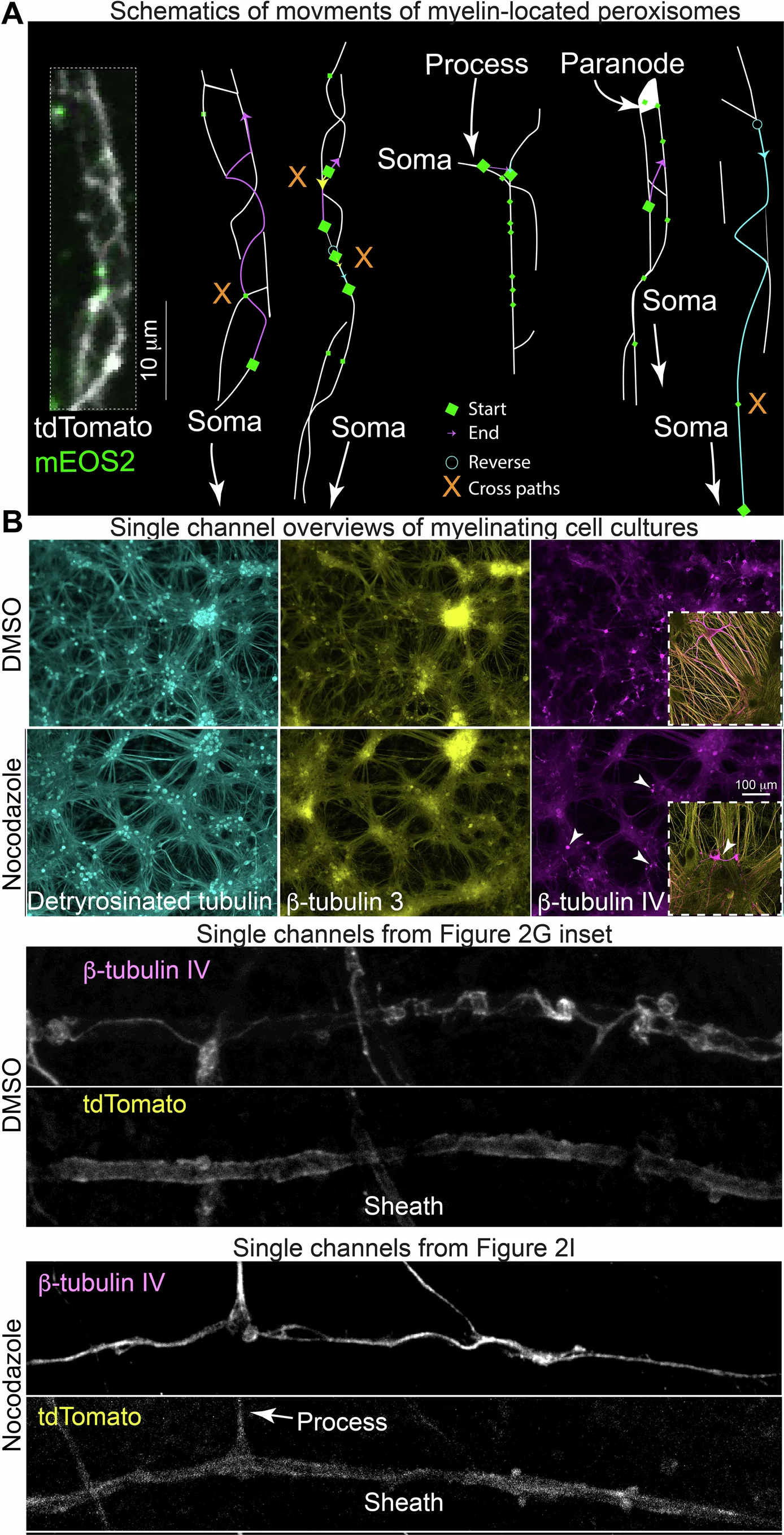
Myelinic peroxisomes are motile and nocodazole partially impairs the microtubule polymer in myelinating cells cultures. **A**. Traces of tdTomato-rendered sheaths (white) and peroxisomes within them (green diamonds) from 10-minute timelapse images from a single z-plane using a spinning disc confocal microscope. On the left, the inset shows a maximum intensity projection (MIP) of a confocal z-stack of the sheath illustrated on its right. Peroxisomes whose paths are shown are indicated by a large green diamond at the start of the movement; those that remain stationary, are indicated by small green diamonds. Magenta arrows show the paths of peroxisomes away from the cell body; yellow arrows show paths to the cell body; and cyan arrows show paths taken by peroxisomes that reverse direction. Arrowheads represent the final destinations within the timeframe. Motile peroxisomes often cross paths with stationary ones (orange Xs) or reverse their direction of travel (cyan circles). See also Supplementary Videos 3, 4, 5 and 6. **B**. Fluorescence micrographs of microtubules in myelinating cell cultures treated with vehicle or 20 μM nocodazole for 3 hours, following extraction of free tubulins. Cells were labeled with anti-detyrosinated tubulin (cyan), a marker of ‘stable’ microtubules; β-tubulin 3 (yellow), which is specific in the CNS to neurons and their processes; and β-tubulin IV (magenta), which is specific in the CNS to oligodendrocytes (white arrowheads), including myelin sheaths. In these cell dense cultures, incubation with nocodazole does not fully perturb the microtubule polymer as can be observed in the images on the left of detyrosinated microtubules and the insets on the right where many β-tubulin 3 and IV containing microtubules appear intact. Overall, however, β-tubulin 3 labeling appears less well^−^defined in the nocodazole-treated cultures compared to the vehicle treated controls (middle panels and insets on the right) due to nocodazole’s interference with microtubule polymerization. The lower panels illustrate the individual color channels from Fig. 2d inset and 2 F, demonstrating that many β-tubulin IV containing microtubules are resistant to nocodazole under the conditions used (20 µM, 3 hours) to treat these cell dense cultures.

The optic nerve stimulation experiment suggests that myelin-located organelles are motile. To explore further, we used live imaging of mEos2-PTS1 oligodendrocyte peroxisomes in myelinating cell cultures. As in other mammalian cell types^29^, most myelin peroxisomes seemed stationery or underwent only Brownian-like motion; however, within a 10-min imaging window, 16 ± 5.4% of myelin-located peroxisomes exhibited movement (distances ≥3 μm; Fig. 2a–d) covering distances up to ~44 µm. Travel was often punctuated by periods of stasis. Some peroxisomes traveled predominantly in one direction (Fig. 2a–c), whereas others reversed the direction (Extended Data Fig. 2a), such that their displacement was less than the distance traveled. Some crossed paths with another (Extended Data Fig. 2a). Often, peroxisomes in the outer tongue traveled toward the paranode (Fig. 2b,c and Supplementary Videos 3 and 4). In Supplementary Videos 5 and 6a, a peroxisome travels to the paranodal loops of myelin via the presumptive outer tongue, traverses four paranodal loops and travels back in the direction of the cell body via the presumptive inner tongue, demonstrating a direct route between inner and outer tongues.

In other eukaryotic cell types, peroxisomes are transported on microtubules^29^. To determine whether this is also true in myelin, we treated myelinating cell cultures with nocodazole, which interferes with the polymerization of microtubules^30^. In these cell dense cultures, 3 h in 20 μM nocodazole led to only minor visual alterations in anti-β-tubulin III staining (Extended Data Fig. 2b), indicating that neuronal microtubules were only partially disrupted. Furthermore, detyrosinated tubulin, a non-cell-type-specific marker of stable microtubules, seemed similar to controls (Extended Data Fig. 2b).

In oligodendrocytes and myelin, anti-β-tubulin IV stained continuous structures within myelin’s cytoplasmic spaces in 94± 2.0% of tdTomato-positive oligodendrocytes (Fig. 2d, Extended Data Fig. 2b and Supplementary Video 7; *n* = 33 cells; two independent cell cultures), but in only 45 ± 7.0% of cells after nocodazole (Fig. 2e,f and Extended Data Fig. 2b; *n* = 17 cells; two independent cell cultures). Notably, in both conditions, the amount of polymerized β-tubulin IV varied considerably from sheath to sheath and from cell to cell.

By live imaging of mEos2-PTS1-labeled peroxisomes, nocodazole caused a significant reduction in the percentage of motile myelin-located peroxisomes (Fig. 2g) without significantly altering distance, displacement or speed (Fig. 2h–j). Nonetheless, the maximum distance traveled by a single peroxisome in 10 min was reduced from ~44 μm in controls to ~17 μm in nocodazole-treated cells (Fig. 2h).

In summary, myelin’s cytoplasmic spaces harbor an active, microtubule-dependent transport of peroxisomes, and presumably other membrane-bound cargoes.

### Myelin harbors tubulin modifiers, microtubule-binding proteins and molecular motors

**Extended Data Fig. 3:**
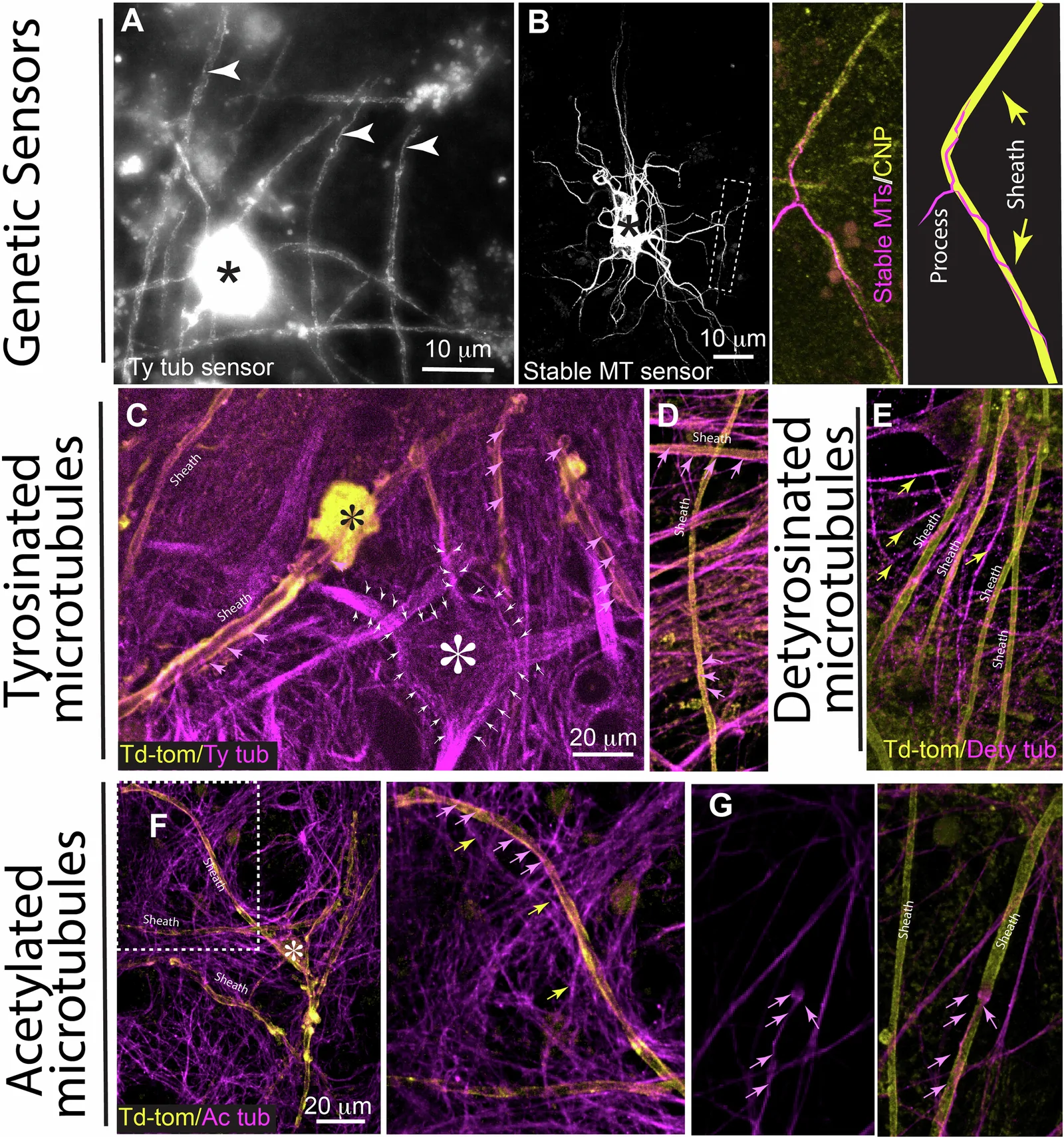
Microtubules in the myelinic channel system harbor various post-translational modifications. **A** and **B**. Genetic sensors labeling tyrosinated tubulins (Ty tub; A) and stable microtubules (stable MTs; B) in myelin, nine hours after transfection. In A, arrowheads point to continuous-appearing structures suggesting these are microtubule polymers. In B, the cell body labeling is saturated, and processes are intensely labeled therefore a higher magnification view of an associated myelin sheath (delineated by the broken line), is shown on the right, stained with anti-CNP and illustrated for clarity in the schematic. The abnormal appearance of the somata and processes is likely because StableMARK itself stabilizes microtubules if overexpressed. **C-F**. Following extraction of free tubulins, immunocytochemistry was used to identify post-translational modifications (PTMs) on microtubule polymers in myelin. Anti-tyrosinated tubulin labeled some myelin sheaths (magenta arrows) of tdTomato-labeled cells, and the cell bodies (asterisk) and process In general, anti-tyrosinated tubulin only partially labeled sheaths (magenta arrows; D). Anti-detyrosinated tubulin did not colocalise with tdTomato positive sheaths but labeled other cell processes (yellow arrows). Where magenta and yellow coincide, the tubulin stain does not follow the contours of the sheath, suggesting it is the processes of overlapping cells (**E**). Anti-acetylated tubulin labeled a few myelin sheaths (magenta arrows) although it was prevalent in other cellular processes (yellow arrows). The oligodendrocyte soma is labeled with an asterisk in the overview image on the left (**F**). Anti-acetylated tubulin only partially labeled myelin sheaths (G).

Our data suggest that myelin contains mixed populations of ‘dynamic’ and ‘stable’ microtubules^31^; stability being encoded in post-translational modifications (PTMs) of tubulins. Indeed, using genetic sensors and immunocytochemistry, we found PTMs on myelin microtubules compatible with this suggestion (Extended Data Fig. 3).

Together these data demonstrate tubular tracks within myelin’s cytoplasmic spaces are required for active translocation of organelles. Interrogation of our databases of purified myelin proteins, identified by label-free mass spectrometry^32^, provided evidence for the presence in myelin of molecular motor proteins, including dynein and kinesin (Supplementary Table 1a). Tubulins, microtubule-binding proteins and tubulin modifiers were also identified (Supplementary Table 1b).

### Neuronal activity influences the motility of myelin peroxisomes

**Fig. 3:**
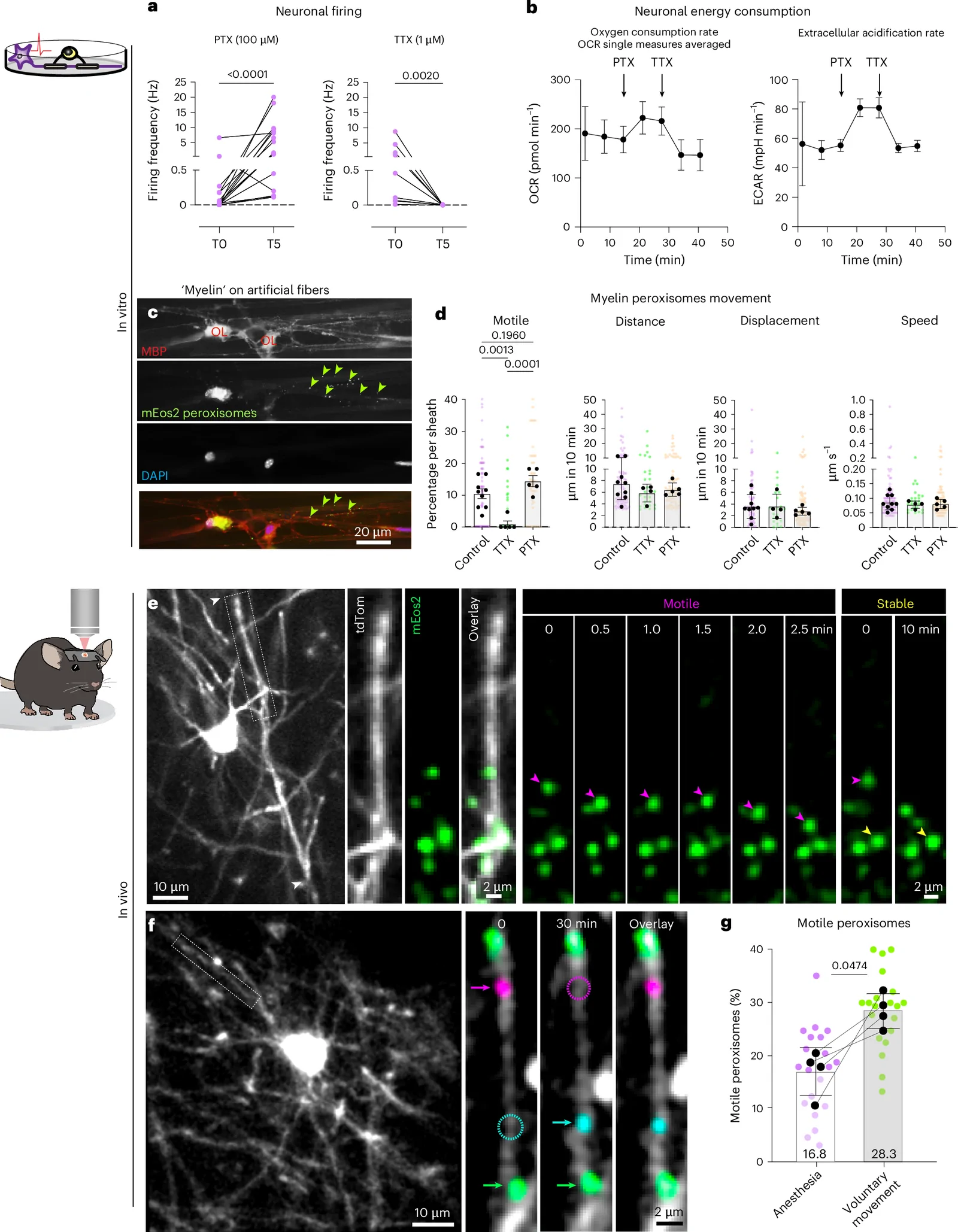
Neuronal activity locally modulates the movement of myelin peroxisomes in vitro and in vivo. **a**, In myelinating cell cultures, PTX or TTX enhance or block neuronal activity, respectively. Graphs show firing frequency of individual neurons recorded in current clamp at baseline (T0) and 5 min (T5) after the application of PTX (100 µM) or TTX (1 µM). A one-sided Wilcoxon matched-pairs signed-rank test was performed; *P* values were obtained from comparison of *n* = 17 or 9 cells, respectively (nine or five independent cell cultures). **b**, In neuron-enriched cultures, oxygen consumption rate (OCR) and extracellular acidification rate (ECR) changed in response to PTX (100 µM) or TTX (1 µM) indicating a general increase and decrease, respectively, in energy consumption. Symbols represent mean ± s.d. (*n* = 3 independent experiments). **c**. Image of peroxisomes (green arrows) in the MBP-positive myelin-like structures formed on inert nanofibers, demonstrating that neuronal activity is not necessary for peroxisomes to enter the myelin sheath during myelinogenesis. See also Supplementary Video 9. **d**, Graphs showing the percentage of myelin peroxisomes per sheath that are motile, distance traveled, distance displaced and velocity when motile. TTX significantly reduced peroxisome motility in comparison to control and PTX-treated cultures (*P* values indicated). PTX did not significantly alter peroxisome motility in comparison to controls (*P* > 0.05). Neither drug significantly altered distance, displacement or speed (*P* > 0.05). Colored dots represent individual sheaths/peroxisomes, black dots represent independent experimental medians. Bars represent mean of independent experimental median ± s.d. See also Extended Data Fig. 4c,d for summed values per experiment. Data were analyzed using a ANOVA followed by Tukey’s multiple comparisons test of independent experimental medians. **e**, Frames from a 20-min timelapse image of a tdTomato-rendered myelin sheath containing mEos2-PTS1-labeled peroxisomes, from the oligodendrocyte shown on the left (individual internode outlined and designated via white arrowheads), in layer 1 of the forelimb region of the primary motor cortex. As in vitro, only a proportion of myelin peroxisomes were motile (magenta arrowheads), while many remained stationery (yellow arrowheads). **f**, Myelin peroxisome location was captured (*z*-stacks) at the start and again at the end of 20-min imaging periods while the animal was walking on a treadmill or anesthetized. The peroxisome(s) indicated in magenta and cyan changed location between the start and end of the experiment while those indicated in green were found in the same location at both time points. **g**, The proportion of myelin peroxisomes that changed location between start and end of imaging was significantly increased when mice walked on the treadmill compared to being anesthetized (*P* value indicated). The same cells were imaged under both conditions (paired data, indicated by gray lines). Colored dots represent individual cells, black dots represent independent experimental medians (*n* = 4 mice). A two-tailed, paired Student’s *t*-test was used to compare independent experimental medians. Bars represent mean of experimental median ± s.d. Source data

Myelin-associated peroxisomes are essential for the long-term integrity of axons, particularly faster spiking axons^33^. Furthermore, axo-myelin glutamatergic signaling and axonal electrical activity trigger neuroprotective exosome release and oligodendroglial metabolic support^15^. To examine the response of myelin-associated peroxisomes to changes in neuronal firing, we used myelinating cell cultures in which neuronal electrical activity and neuronal energy consumption can be enhanced or diminished respectively, with the GABA receptor blocker picrotoxin (PTX; 100 μM) or tetrodotoxin (TTX; 1 μM) (Fig. 3a,b).

**Extended Data Fig. 4:**
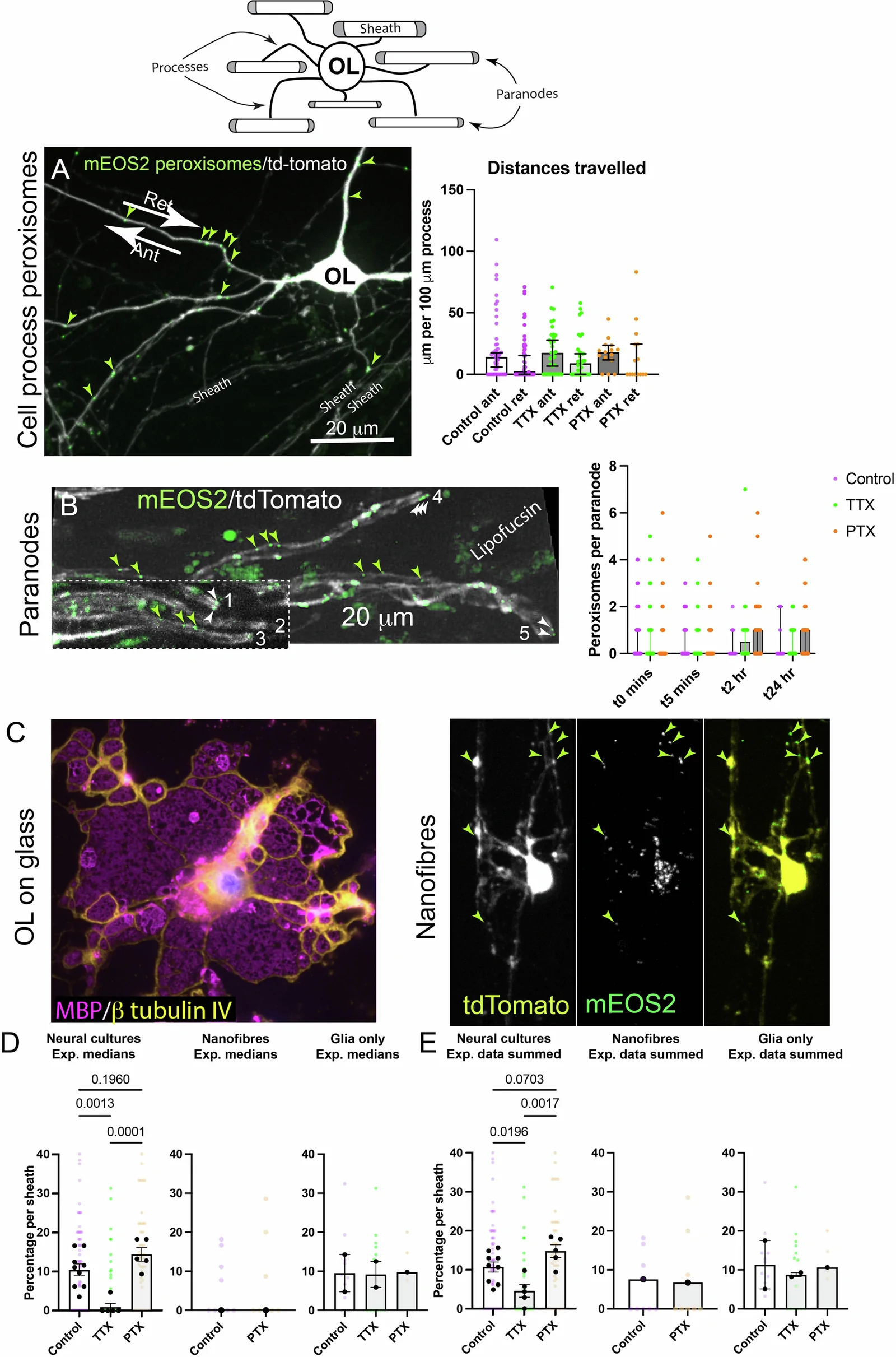
The motility of myelin-located peroxisomes is modulated by neuronal activity but localization and transport to and from the soma is not. The schematic (top) provides orientation for the data reported in A. **A**. Maximum intensity projection (MIP) of tdTomato-labeled oligodendrocyte with green arrows denoting mEOS-PTS1-labeled peroxisomes in processes. In the graph, colored dots represent individual peroxisomes; bars represent medians +/- 95% CI. Modulators of neuronal activity did not significantly alter the distances traveled by motile peroxisomes, in either anterograde or retrograde directions in relation to the cell body. See also Video S7. **B**. The images show two MIPs of sheaths with paranodal endings containing no (paranode 2), one (paranode 3), two (paranodes 1 and 5), or three (paranode 4) peroxisomes. The graph shows the number of peroxisomes per paranode (colored dots) in control, TTX (1 µM) or PTX (100 µM) treated cultures at 5 minutes, 2 hours or 24 hours post treatment. Bars represent median values +/- 95% CI. The number of paranodes examined per treatment and time point ranged from 10 to 69 (1–5 independent experiments). Statistical analyses was performed using data from individual paranodes. Data were analyzed using a two-way ANOVA, mixed effects model. No significant differences were observed between conditions or time points. **C**. Widefield fluorescence microscopy image of an MBP and β−tubulin IV labeled olidodendrocyte grown on glass (LHS) and maximum intensity projection of a confocal image of a tdTomato positive oligodendrocyte soma connected by cellular processes to two parallel myelin sheaths, inside which a number of peroxisomes can be observed (indicated by green arrowheads). The same cell is illustrated in Supplementary Movie 8. **D**. Graphs showing that the motility of myelin-located peroxisomes is modulated by TTX in neural cell cultures as shown also in Fig. 3. When ‘myelin’ is wrapped around artificial axons (nanofibers), the proportion of myelin peroxisomes that were motile is similar to that observed when neural cell cultures were treated with TTX. In glial cell cultures, where oligodendrocytes extend a myelin-like membrane over the glass coverslip, the proportion of motile peroxisomes was not modulated by TTX or PTX. Black dots represent experimental medians, colored dots represent values from individual sheaths/cells. Bars represent the mean of the experimental medians +/- sem. **E**. The same data as in A, but here the black dots represent the percentage value obtained by summing the total number of myelin peroxisomes and the total number of motile myelin peroxisomes quantified in each independent experiment.

Live imaging of oligodendrocyte processes over 10 min (beginning 5 min after drug) showed that modulating neuronal activity had no effect on retrograde or anterograde transport of peroxisomes between the sheath and the soma (Extended Data Fig. 4a and Supplementary Video 8). This is consistent with peroxisomes entering ‘myelin’ formed on inert nanofibers (Fig. 3c). Nonetheless, in the presence of neurons, the proportion of myelin peroxisomes that were motile was significantly reduced in TTX compared to control (Fig. 3d). Despite this, distance, displacement and velocities were not significantly altered (Fig. 3d). PTX did not significantly alter any of the parameters measured, although we observed an upward trend in motility and a downward trend in displacement (Fig. 3d). We found no evidence that the number of peroxisomes at paranodes, where myelin is tethered to the axon, changed in response to TTX or PTX (Extended Data Fig. 4b).

We repeated drug treatments on murine spinal cord-derived glial cell cultures (astrocytes, oligodendroglia and microglia but not neurons) grown on glass or on artificial axons (Extended Data Fig. 4c). We reasoned that if the effect on motility was truly mediated by neuronal activity, peroxisome motility on inert axon-like nanofibers would seem similar to that in TTX-treated neural cell cultures. Whether examining the experimental median (Extended Data Fig. 4d) or the sum of individual values (Extended Data Fig. 4e), our data support this suggestion (Supplementary Video 9). In flattened oligodendrocytes on glass, peroxisome motility (assessed in secondary processes) was higher than on nanofibers and was unaltered by TTX or PTX compared to controls, suggesting these agents do not alter oligodendroglial peroxisome motility directly or via effects on other glia.

To determine the in vivo relevance of our observations, we next examined the movement of myelin peroxisomes in the forebrain motor cortex of live mice. Myelin sheaths were imaged in the forelimb region of the motor cortex of mice with a two-photon microscope during voluntary ambulation on a treadmill and subsequently when anesthetized with isoflurane (both, 20-min timelapse imaging; Supplementary Video 10). As in vitro, most myelin peroxisomes were stationary (yellow arrows, Fig. 3e) whereas ~20% were motile (magenta arrows, Fig. 3e) in awake mice. Volumetric *z*-stacks were taken at start and end of each 20-min period to compare the localization of individual peroxisomes across imaging periods (Fig. 3f). Of note, the fraction of peroxisomes that changed location was increased to ~30% following treadmill ambulation (Fig. 3g). While isoflurane has been associated with mitochondrial complex-I inhibition, mature oligodendrocytes are glycolytic cells with severely reduced OXPHOS^11^. Moreover, physiological ATP levels are not rate-limiting for motor proteins on microtubular tracts^34^.

Together, these data suggest that neuronal activity influences myelin’s transport processes locally, at the level of the sheath.

### Oligodendrocytes require KIF21B to maintain axon integrity

**Extended Data Fig. 5:**
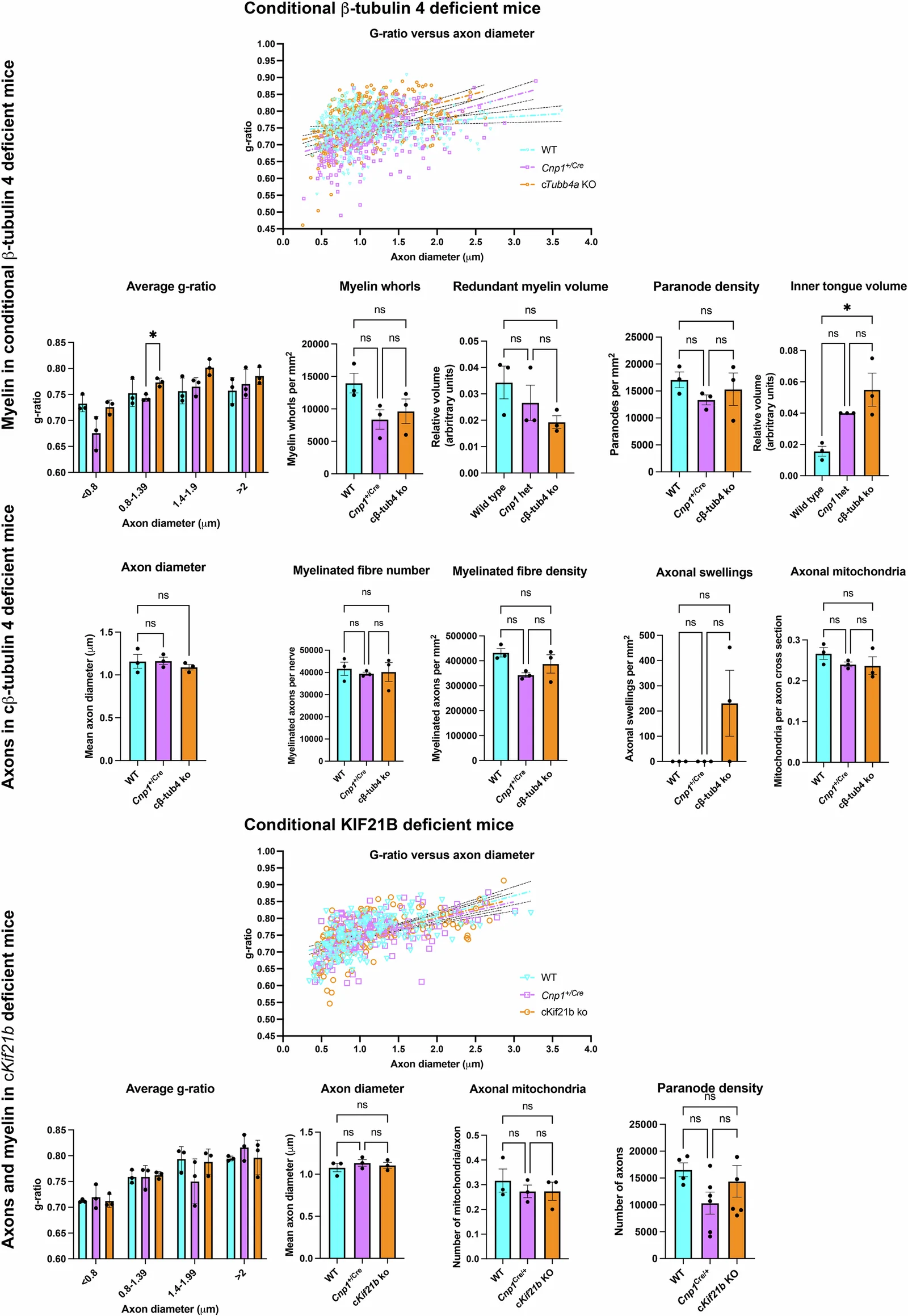
Morphometry of optic nerve of conditional β-tubulin 4 and conditional KIF21B deficient mice. **Upper** Quantification of electron micrographs of optic nerve fibers of 14-month-old *Tubb4a*^*flox/flox*^*;Cnp*^*+/+*^ (WT), *Tubb4a*^*+/+*^*;Cnp*^*+/Cre*^ *(Cnp1*^*+/Cre*^*) and Tubb4a*^*flox/flox*^*;Cnp*^*+/Cre*^ (cβ-tub4 ko) showed that in axons larger than 0.8 mm diameter, myelin tended to be thinner in the conditional knockout (higher g-ratio) compared to controls, but the differences were mainly not significant. For g-ratio versus axon diameter, data were analyzed using a 2-way ANOVA (p = 0.0303 for g-ratio x genotype) followed by a Tukey’s multiple comparisons test. For myelin whorls, redundant myelin, paranodes, inner tongue volume, axon number, axon density, axonal swellings, axonal mitochondria, data were analyzed using an ordinary one-way ANOVA (p = 0.1128, p = 0.2280, p = 0.4688, p = 0.0132, p = 0.8698, p = 0.0993, p = 0.1177. p = 0.3852, respectively). A Tukey’s multiple comparisons test showed inner tongue volume was significantly higher in the cβ-tub4 ko than in the WT control but not significantly different from the *Cnp1*^Cre/+^ control suggesting CNP haploinsufficiency is the main contributor to inner tongue enlargement (p = 0.0113). **Lower** Quantification of electron micrographs of optic nerve fibers of 12-month-old *Kif21b*^*flox/flox*^;*Cnp*^*+/+*^ (WT), *Kif21b*^*+/+*^;*Cnp*^*+/Cre*^ *(Cnp1*^*+/Cre*^*) and Kif21b*^*flox/flox*^*;Cnp*^*+/Cre*^ (c*Kif21b* ko) showed no significant change in g-ratio across various axon diameters, data were analyzed using a 2-way ANOVA (p = 0.0578 for g-ratio x genotype). For axon diameter, paranode density and axonal mitochondria, data were analyzed using an ordinary one-way ANOVA (p = 0.6594, p = 0.6583, p = 0.2337, respectively). Source data

These data led us to hypothesize that interfering with microtubules in oligodendrocytes would have secondary consequences for maintenance of the myelinated axon. In the CNS, β-tubulin IV is enriched in oligodendrocytes and localizes to the somata, processes and myelin^13,35^, leading us to hypothesize that β-tubulin IV could have a role in cargo transport, sheath formation and/or axon maintenance; however, targeting the *Tubb4* gene selectively in oligodendrocytes in *Tubb4*^*fl/fl*^::*Cnp*^+/*cre*^ mice did not lead to overt changes in myelinated CNS fibers, even at 14 months of age, except that the g-ratio tended to be slightly higher than in controls (Extended Data Fig. 5a). Thus, *Tubb4* function is likely compensated by other β-tubulins.

**Fig. 4:**
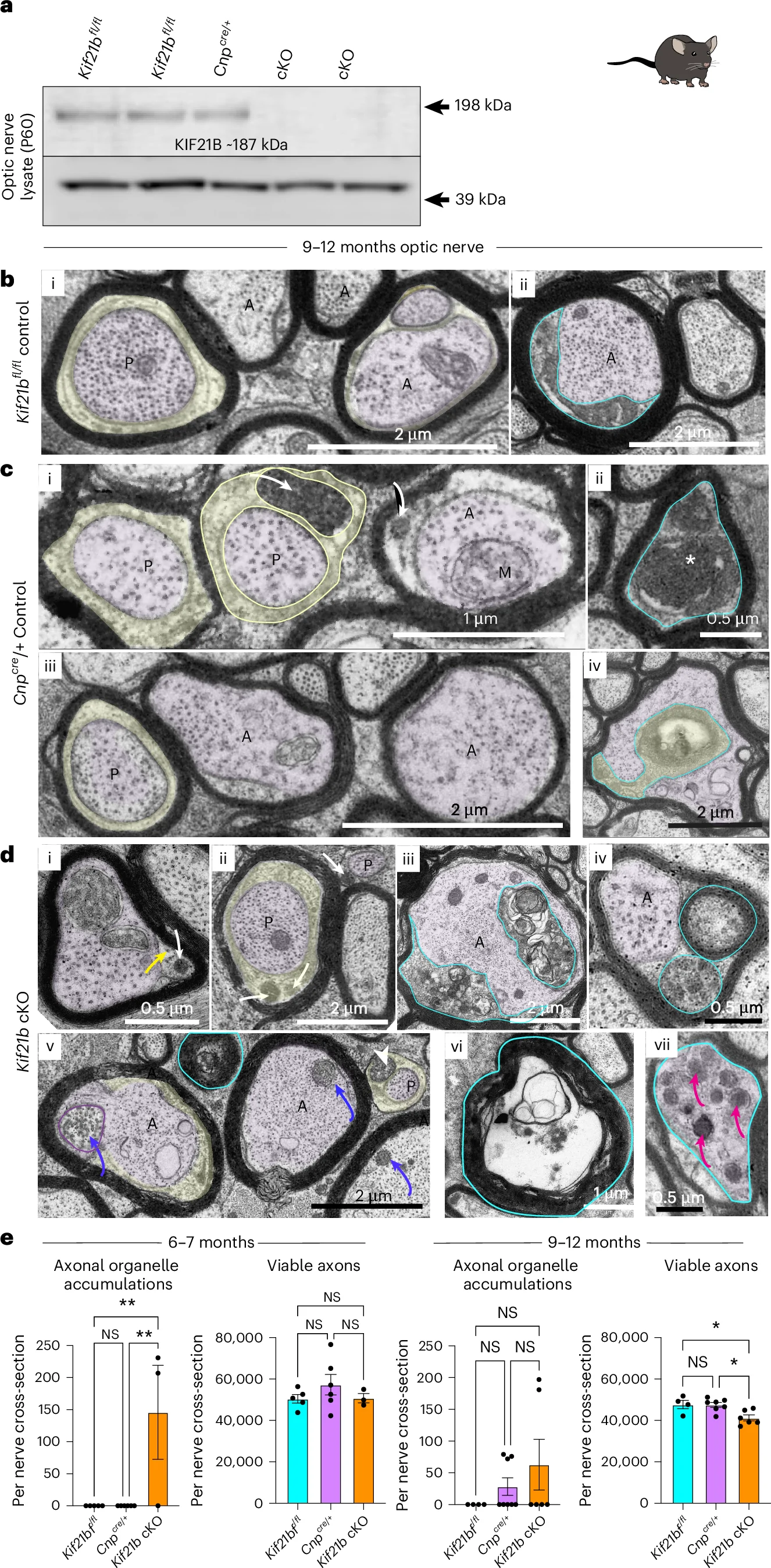
Oligodendrocytes require KIF21B to maintain axon integrity. **a**, In the optic nerve, a pure white matter tract lacking neuronal cell bodies and dendrites, *Cnp*^*c*^^*re*^-dependent deletion of *Kif21b* led to depletion of KIF21B in whole nerve lysate, demonstrating this motor protein is expressed in oligodendrocytes (data from two independent litters). **b**, Electron micrographs of optic nerve axons (A) from 9–12-month *Cnp*^+/+^::*Kif21b*^*fl/fl*^ controls. Note the exceptional finding of periaxonal electron dense material (blue outline, ii). Paranodes (P) were present on ~3.22 ± 0.64% of myelinated axons. Electron dense material was observed too sporadically to quantify accurately. **c**, In *Cnp*^*cre*/+^ control optic nerves, myelinated axons seemed largely normal. Periaxonal electron dense organelles (i) (white arrows) are also seen in wild-type (WT) mice (Fig. 1). Rarely, axons seemed electron dense and degenerating (asterisk in ii) or myelin’s cytoplasmic spaces were abnormally extended (blue outline in iv). **d**, In *Kif21b* cKO, most myelinated axons seemed morphologically normal (i and ii). Myelin organelles were observed in myelin’s inner tongue (i) and paranodal loops (ii) as in WT (Fig. 1). In i, the yellow arrow points to a microtubule. Myelin’s cytoplasmic spaces were occasionally locally enlarged, impinging on the axon (delineated in blue in iii). Redundant myelin and/or abnormal electron dense material were observed in some swollen inner tongues (outlined in blue in iv). Extracellular myelin whorls (blue outlines in v and vi) were not more frequent than in controls. Rarely, axons contained abnormal-appearing mitochondria (M; blue arrow on the left in v in comparison to the two normal-appearing mitochondria indicated by blue arrows on the right). Axonal organelle accumulations were also observed in *Kif21b* cKO mice (magenta arrows in vii.) **e**, Quantification of axonal organelle accumulations and total axon numbers in *Kif21b* cKO optic nerves and controls at age 6–7 (left) and 9–12 months (right). Black dots represent independent experimental mean values, each derived from a single optic nerve per animal. Data were analyzed by one-way ANOVA followed by Tukey’s multiple comparisons test. *P* values indicated on graphs. Bars represent mean ± s.d. Source data

Next, we turned to a motor protein, KIF21B that we identified as a putative candidate for a function in myelin’s cytoplasmic spaces because polymorphisms in *KIF21B* are associated with increased susceptibility to multiple sclerosis (MS). Moreover, *KIF21B* is upregulated ~tenfold in MS white matter^36,37^, and expression of *Kif21b* is differentially regulated in different populations of mature oligodendrocytes in mouse spinal cord of experimental autoimmune encephalomyelitis^38^. KIF21B is both a plus-end-directed processive motor protein and a modulator of microtubule dynamics^22^, which in neurons is enriched in dendrites and growth cones at neurite tips^39^. To determine whether KIF21B is expressed in oligodendrocytes in the optic nerve, we compared by western blotting, optic nerve lysates from *Kif21b* conditional knockout (*Kif21b* cKO) mice (*Cnp*^*cre*/+^::*Kif21b*^*fl*/*fl*^) with two controls (*Cnp*^*+*/+^::*Kif21b*^*fl*/*fl*^ and *Cnp*^*+*/*cre*^::*Kif21b*^+/*fl* or +/+^). While *Cnp*^*cre*^ can recombine ectopically, retinal ganglion cells (whose axons traverse the optic nerve) do not recombine, and in the optic nerve, all recombined cells are oligodendrocytes^40^. KIF21B was below detection levels by western blotting of whole optic nerve lysates from *Kif21b* cKO mice but was readily detectable in control optic nerves (Fig. 4a), demonstrating that, in this white matter tract, KIF21B is predominantly expressed by *Cnp*-expressing oligodendrocytes.

To determine whether ablating KIF21B from oligodendrocytes had secondary consequences for the myelinated axon, we examined optic nerve cross-sections by TEM (Fig. 4b–d). Across all genotypes, most myelinated axons seemed morphologically normal at 1 year of age (Fig. 4b(i),c(i),(iii),d(i),(ii))). Accordingly, the g-ratio, axon diameter, axonal mitochondrial numbers and paranodal densities were unaltered across the three genotypes at 9–12 months of age (Extended Data Fig. 5b); however, in *Kif21b* cKO mice, inner tongues sometimes seemed distended and contained membranous material (Fig. 4d(iii),(iv)) and occasionally, otherwise healthy-appearing axons contained mitochondria lacking normal-appearing cristae (blue arrows, Fig. 4d(v)). Accumulations of axonal organelles (magenta arrows, Fig. 4d(vii)), indicative of axonal transport stasis, were observed in *Kif21b* cKO mice at 6 months (Fig. 4e) and also at 4 months of age but were largely ‘lost’ by age 9–12 months, suggesting these pathologically affected axons had degenerated at this age (Fig. 4e).

While similar pathological axonal changes were seen occasionally also in heterozygous *Cnp*^+/*cre*^ controls at 9–12 months of age (Fig. 4c(ii)), there was no comparable loss of optic nerve axons (Fig. 4e), suggesting that KIF21B-dependent transport and/or microtubule destablization in oligodendrocytes are required for long-term axonal integrity.

### A role for CNP and KIF21B in maintaining monocarboxylate transporter levels

**Fig. 5:**
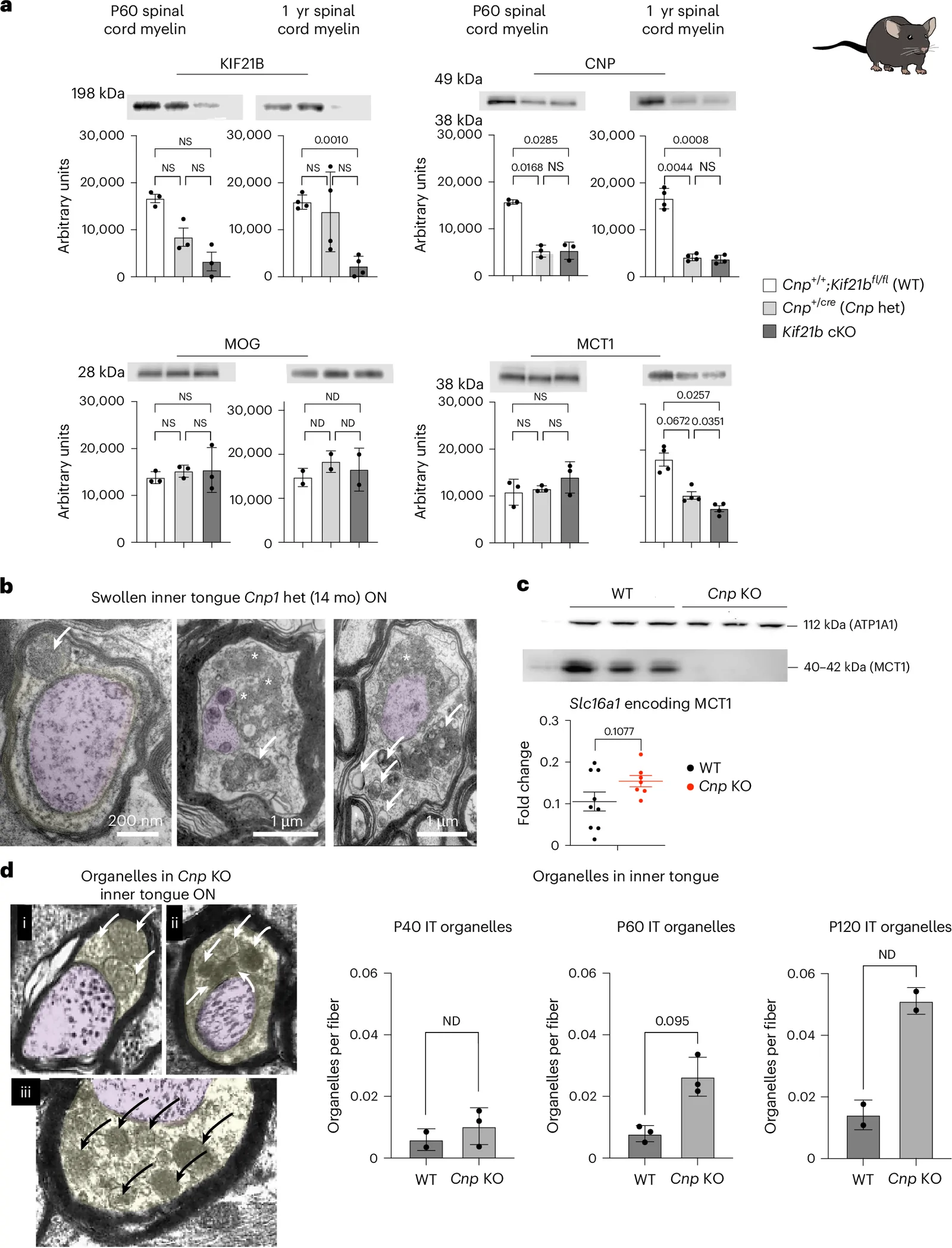
Monocarboxylate transporter 1 is reduced when intramyelin transport is impaired. **a**, Western blots of spinal cord myelin-enriched fractions of P60 and 1-year-old mice. Levels of KIF21B were reduced in *Kif21b* cKO mice compared to *Cnp*^+/+^::*Kif21b*^*fl*/*fl*^ WT controls, albeit that the reduction was not significant at P60 due to intra-group variation. Unexpectedly, levels in the *Cnp*^*cre*/+^ controls were highly variable. Levels of CNP were significantly reduced in both the *Cnp*^*cre*/+^ and *Kif21b* cKO mice compared to the control, at P60 and 1 year, as expected. MOG levels were similar across genotypes at both ages. At P60, MCT1 levels were similar across genotypes, but at 1 year of age, levels were nonsignificantly reduced in the *Cnp*^*cre*/+^ mouse compared to WT control and significantly reduced in the *Kif21b* cKO compared to both WT and *Cnp*^*cre*/+^ controls. Data were analyzed using a one-way ANOVA followed by Tukey’s multiple comparisons test (P60, *n* = 3; 1 year, *n* = 4). Black dots each represent independent mouse spinal cords. Bars represent mean ± s.d. ND, not determined; NS, not significant. **b**, Electron micrograph (EM) of myelinated optic nerve (ON) axons from heterozygous *Cnp*^+/*cre*^ mice at age 14 months. Normal-appearing paranode containing a peroxisome-like organelle (white arrow) (left). Membranous structures (white arrows) and dense cytoplasm (asterisks) seem to accumulate in inner tongues in a small number of fibers (right). Inner tongues seem grossly swollen and impinge upon the axon. These were observed too sporadically to quantify accurately; approximately 4 per 50-nm thick optic nerve cross-section, comprising ~50,000 axons. **c**, Western blots of myelin-enriched fraction from homozygous *Cnp* knockout (KO) mice shows a dramatic reduction in steady-state levels of MCT1. By contrast, RT–PCR of brain lysates demonstrates no significant change in *Slc16a1* mRNA, when examined in relation to *Slc16a1* mRNA in myelin from P20 WT mouse brain, in agreement with a post-transcriptional MCT1 deficit. Data were analyzed using a two-tailed, unpaired Student’s *t*-test. Each dot represents an independent animal. Lines represent mean ± s.e.m. **d**, EMs of myelinated optic nerve axons from homozygous *Cnp* KO mice, taken at (i) P40, (ii) P60 and (iii) P120, with undefined organelles (white arrows) residing in the inner tongues (yellow overlay) (left). Quantification demonstrates these organelles accumulate from age P60, which is long before clinical symptoms emerge at age 6 months^23^. P60 data were analyzed using a two-tailed, unpaired Student’s *t*-test. Data points represent one independent optic nerve (one nerve per animal was examined). Bars represent mean ± s.d.; *n* < 3. Source data

**Extended Data Fig. 6:**
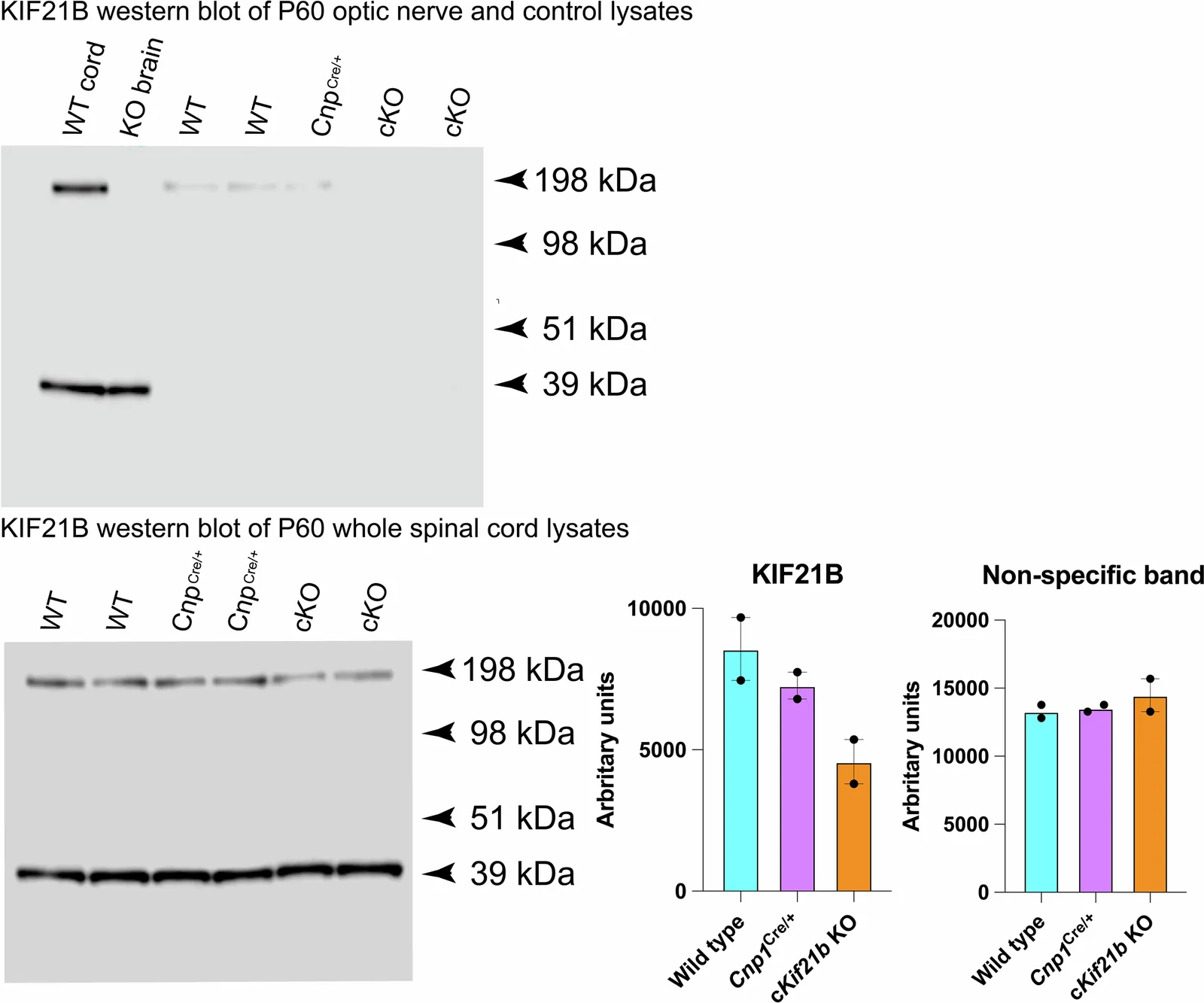
Western blotting of whole optic nerve and spinal cord lysates indicates KIF21B is enriched in oligodendrocytes, in addition to neurons. In western blots of optic nerve (right hand side of upper panel) which does not contain neuronal cell bodies or dendrites, KIF21B is markedly diminished in the *Kif21b* conditional knockout mice (cKO) in comparison to *Kif21b*^*flox/flox*^*;Cnp*^*+/+*^ (WT) and *Kif21b*^*+/+*^*;Cnp*^*+/Cre*^ *(Cnp1*^*+/Cre*^*) controls*. This indicates KIF21B is enriched in *Cnp1*-expressing cells (oligodendrocytes) in this white matter tract. Whole spinal cord lysate from a wild-type mouse and brain from a global knockout mouse were run alongside to confirm antibody specificity. In western blots of whole spinal cord lysates (lower panel), KIF21B was only slightly diminished in the c*Kif21b* KO, as expected for a protein that is expressed in both neurons and oligodendrocytes. A band at ~39 kDa, due to secondary antibody binding non-specifically in cord and brain lysates, confirms equal loading of samples. Bars represent mean ± SD. Data points from individual mice.

Transport processes in myelin’s cytoplasmic spaces could contribute to overall myelin protein turnover. To determine if oligodendroglial KIF21B depletion impacts specific protein levels in myelin, we performed western blotting of spinal cord myelin-enriched fraction from mice at postnatal day 60 (P60) and 1 year. As expected, KIF21B itself was strongly reduced in purified myelin from *Kif21b* cKO mice (Fig. 5a) and to a lesser degree in total spinal cord homogenate containing both white and gray matter (Extended Data Fig. 6); however, the *Cnp*^*cre*^ knock-in allele (resulting in reduced *Cnp* dosage) was likely to contribute to these effects as CNP maintains the integrity of myelin’s cytoplasmic spaces^12^. Indeed, in myelin from 1 year-old *Cnp*^+/*cre*^ littermate controls, KIF21B levels became remarkably variable (Fig. 5a). In comparison, myelin oligodendrocyte glycoprotein (MOG), a transmembrane protein on the outer myelin layer, was unaltered in *Kif21b* cKO mice compared to either control. By contrast, levels of MCT1, which is required at the glial–axonal junction for the transfer of pyruvate/lactate to the periaxonal space^2,3^ were significantly reduced in 1-year-old mutants, but not yet in their P60 counterparts (Fig. 5a). Indeed, MCT1 was also reduced, albeit not significantly (*P* = 0.067), in 1-year-old *Cnp*^*+/cre*^ controls, suggesting that both KIF21B and CNP are required for maintaining normal MCT1 levels at the glial–axon junction. Notably, there was no significant change in the levels of β-tubulin IV in purified myelin from *Kif21b* cKO mice, suggesting no lack of tubular tracks. We thus hypothesize that the loss of myelin’s cytoplasmic space integrity causes a slow attrition in delivery of MCT1 to the glial–axonal junction.

### Accumulation of myelin-located organelles in the absence of CNP

To test our hypothesis further, we compared myelin composition and myelinated fiber morphology in heterozygous (*Cnp*^+/*cre*^) and homozygous (*Cnp*^−/−^) mutants. In optic nerves from *Cnp* heterozygous mice, at 14 months, membranous structures (arrows) and dense cytoplasm (asterisks) were occasionally observed in enlarged inner tongues (Fig. 5b), resembling changes in the aged CNS^41^. In homozygous *Cnp* mutants, axonal changes in myelinated fibers begin as early as P5 (ref. ^18^). By western blotting of control and *Cnp1*^−/−^ myelin-enriched brain fraction, MCT1 was strongly reduced by at least P75, whereas transcription of the encoding gene (*Slc16a1*) was similar in mutants and controls (Fig. 5c), suggesting the absence of CNP affects the delivery of MCT1 to the glial–axonal junction and/or its turnover. In support of the former, we found that organelles seemed trapped within myelin’s cytoplasmic spaces (Fig. 5d) in homozygous *Cnp* mutants.

### Myelin organelle accumulation and axon degeneration in aged white matter

**Fig. 6:**
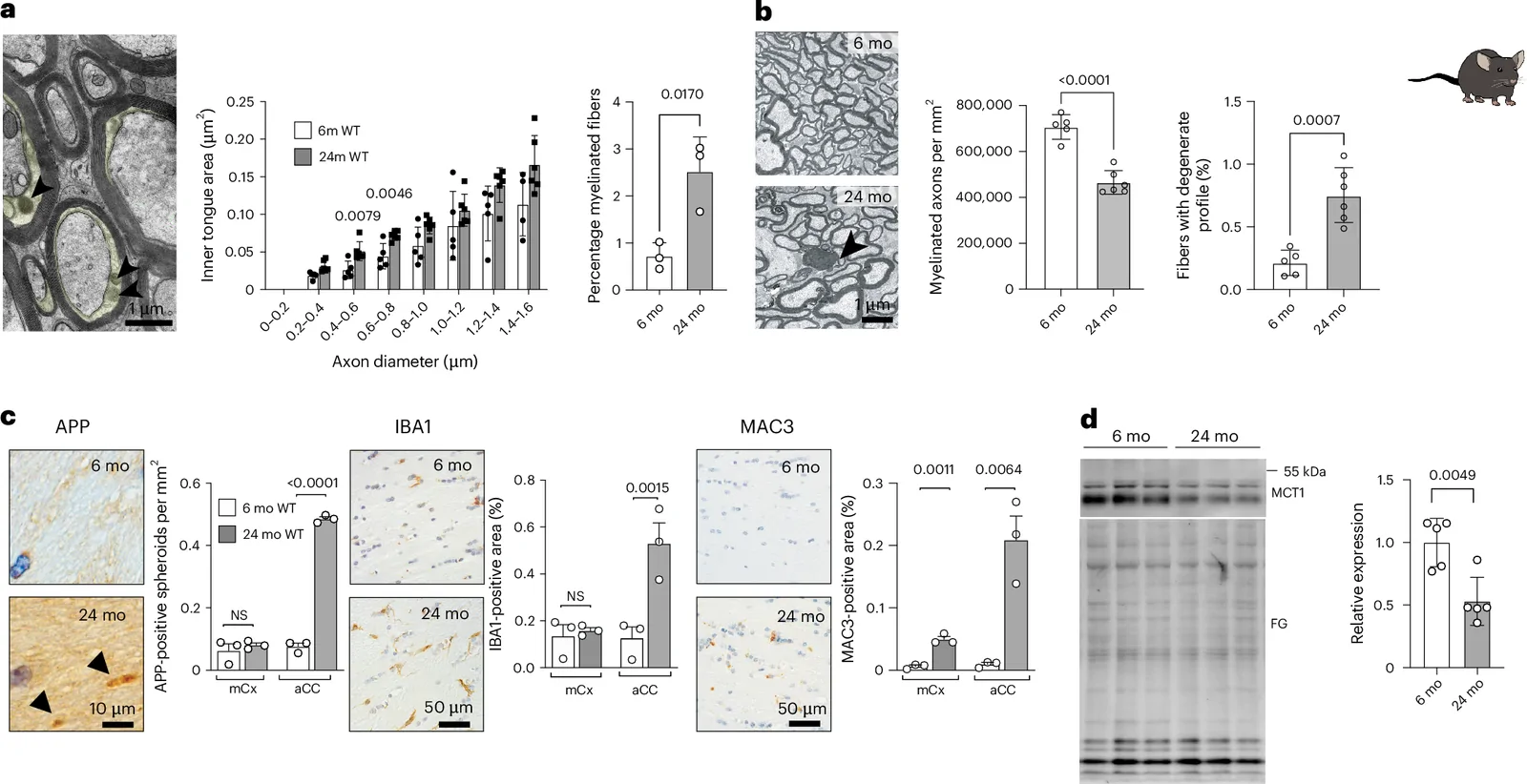
White matter aging leads to changes in myelin’s cytoplasmic spaces, decreased MCT1 levels and axon degeneration. **a**, EM of optic nerves from a 24-month-old mouse preserved by high-pressure freezing (HPF), demonstrating that inner tongues (yellow) are occasionally enlarged and more likely to contain electron dense organelles (black arrows). Inner tongue areas tended to be increased in 24-month-old mice compared to 6-month-old mice across all axon diameters (180–230 axons per nerve were examined), reaching significance in smaller diameter fibers (right). Data were analyzed using multiple unpaired *t*-tests, corrected for multiple comparisons using Holm–Šídák method (*n* = 5 or 6 mice). Higher proportion of myelinated fibers have organelles in the inner tongue (left). Data analyzed using an unpaired two-tailed Student’s *t*-test (*n* = 3 mice). Each dot represents an independent animal. Bars represent mean ± s.d. **b**, EM overview of HPF nerve showing that in 24-month-old WT mice, the density of myelinated axons is significantly reduced compared to 6-month-old mice, and the percentage of fibers with a degenerate profile (black arrow) is significantly increased (860–1,300 axons per nerve were examined). Data were analyzed by unpaired, two-tailed Student’s *t*-tests (*n* = 5 or six nerves). Each dot represents an independent nerve. Bars represent mean ± s.d. **c**, Micrographs and graphs showing that APP-positive axonal swellings and IBA1-labeled microglia are increased in 24-month-old mice compared to their 6-month-old counterparts in the corpus callosum (aCC) but not in the motor cortex (mCx), bregma 0.74 mm. Mac3-positive microglia were significantly increased in both aCC and mCx in aged compared to younger mice. Data were analyzed by unpaired, two-tailed Student’s *t*-tests for each region of interest (*n* = 3 mice per group). Each dot represents an independent animal. Bars represent mean ± s.d. **d**, Western blot showing that MCT1 is about 50% decreased in a ‘myelin-enriched’ brain fraction from 24-month-old mice, compared to 6-month-old mice (top), using fast green (FG) as an internal standard. Data were analyzed by an unpaired, two-tailed Student’s *t*-test (*n* = 5 per group). Each dot represents an independent animal. Bars represent mean ± s.d. Source data

Advanced age is associated with altered integrity of myelin in white matter tracts and intracortical fibers^42,43^ with axon vulnerability as assessed by magnetic resonance imaging (MRI) in humans^44^. We hypothesized that myelin changes include alterations in myelin’s cytoplasmic spaces, similar to its destabilization by loss of CNP. This is suggested by a peculiar interaction of *Cnp* heterozygosity and advanced brain age on behavioral symptoms in mice^45^. To directly determine whether age-related white matter changes impact intramyelin transport processes, we compared myelin morphology and biochemistry in wild-type mice at age 6 and 24 months. We observed a volume increase in inner togue in the aged mice, most notably in small diameter fibers, accompanied by a twofold increase in fibers with organelles in the inner tongue (Fig. 6a). Furthermore, we observed an almost twofold reduction in the overall density of myelinated axons at 24 months (corpus callosum) and a doubling of axonal degeneration profiles (Fig. 6b).

Brain sections stained for amyloid precursor protein (APP), a marker of axonal transport impairment, had a significant increase in APP-positive swellings in the corpus callosum (Fig. 6c), but not the cortex, of 24-month-old mice. These changes were accompanied by increased densities of IBA1 and MAC3-positive microglia/macrophages. Notably, by western blotting of myelin-enriched fractions, steady-state level of MCT1 was approximately 50% reduced in the 24-month-old mice compared to 6-month-old controls (Fig. 6d). Together, these data suggest that aging-associated changes impair normal transport processes in myelin’s cytoplasmic spaces, having a profound impact on MCT1 levels.

## Discussion

**Fig. 7:**
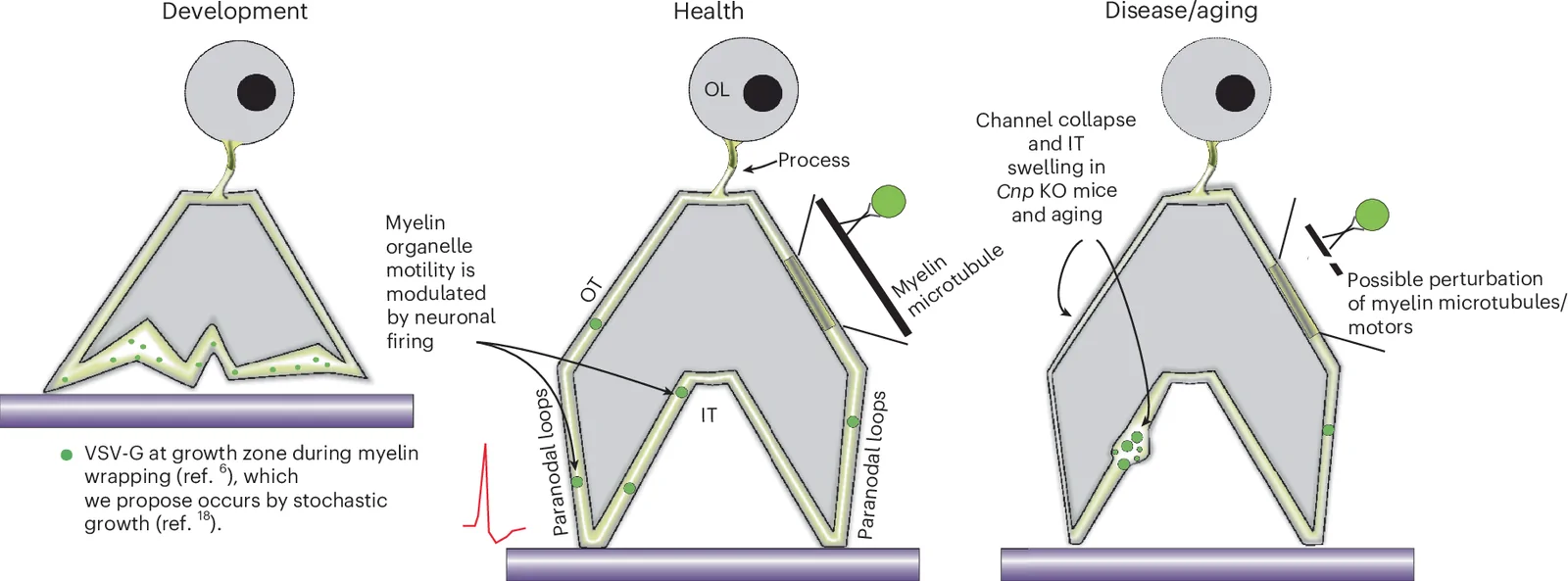
Summary schematic illustrating the unwrapped myelin sheath during development and in the adult, in health and disease. During development, vesicular stomatitis virus glycoprotein (VSV-G) is found at the leading edge (growth zone), suggesting that it is actively transported there^6^. In the mature sheath, when wrapped around the axon, the microtubule-dependent movement of myelin peroxisomes is modulated by neuronal activity. In disease and aging, myelin organelles accumulate in the inner tongue, possibly due to perturbation of myelin microtubules and/or motors. In the *Cnp* knockout mouse model of disease, there is collapse of myelin’s cytoplasmic spaces and swelling of the inner tongue, with or without organelle accumulation^18^. In disease and aging, these changes are associated with decreased levels of MCT1, needed for exchange of monocarboxylates between the sheath and the axon, in biochemically enriched myelin. Together, our data support the importance of myelin’s cytoplasmic spaces for axon integrity.

The physiological function of compacted myelin in axonal insulation and impulse conduction is well established, but a distinct role for ‘noncompacted’ myelin has been obscure. Noncompacted myelin was identified already by light microscopy by Rio de Hortega et al. in 1928^46^ but suspected an experimental artifact (reviewed elsewhere^13^). While microtubules and membranous structures were reported in noncompacted myelin^14^, TEM failed to show whether these are stationary remnants of development, myelin turnover or part of a vital transport system, analogous to axonal transport, between the oligodendrocyte soma and the innermost cytoplasmic myelin layer facing the axon. Here, we establish the concept of motor-driven, microtubule-dependent organelle transport in established myelin sheaths, which we suggest is critical for long-term axonal integrity. Moreover, our observation that neuronal electrical activity modulates myelin transport suggests that myelinated neurons themselves can control, at least in part, aspects of oligodendrocyte behavior (Fig. 7).

Our interest in these phenomena was triggered by the observation that oligodendrocytes and axons are metabolically coupled^2^ through MCT1 at the glial–axonal interface^3^. It is unclear currently whether myelin turnover in adult mice rests entirely on the incorporation of new membranes at the innermost myelin layer^19^. Nonetheless, the necessary turnover of MCT1 and its functional incorporation at the periaxonal myelin membrane would require vesicular transport to the inner tongue. We thus hypothesized that MCT1 levels in mature myelin depend on the integrity of the intramyelin transport route. Our data support this suggestion by demonstrating a severe depletion of MCT1 and increased frequencies of organelles in myelin from CNP-deficient mice in which myelin’s cytoplasmic spaces is structurally impaired^12^, findings replicated in aged animals. In particular, the ‘trapping’ of myelin is reminiscent of organelle accumulations in axons upon axonal transport stasis (reviewed elsewhere^17^).

To model and visualize transport of such nuclear-encoded membrane-bound cargoes in myelin’s cytoplasmic spaces, we chose fluorescently labeled peroxisomes; organelles that are abundant in myelinating glia. While used here as a surrogate for myelin organelles more generally, it is noteworthy that oligodendroglial peroxisomes are essential for long-term axonal integrity^32^.

The existence of a transport system operating in mature fibers is plausible because myelin’s cytoplasmic spaces are developmentally derived from subcellular compartments of oligodendrocytes, in which transport processes contribute directly to myelin outgrowth. Indeed, normal myelin sheath growth in vivo requires nucleation of microtubules, which is mediated by tubulin polymerization promoting protein (TPPP)^47^. Further, mRNAs encoding myelin proteins (Extended Data Fig. 1) and presumably the oligodendroglial translation machinery, are transported on microtubules, as demonstrated in oligodendrocyte monolayer cultures (ref. ^48^ and others).

The importance of transport processes for developmental myelination and myelin maintenance is amply illustrated by the spontaneous *taiep* mutant rat, which harbors a point mutation in *Tubb4a*, encoding β-tubulin IV. This increases the ratio of ‘minus-end-distal’ to ‘plus-end-distal’ microtubules in the fine processes of developing oligodendrocytes^49^, leading to an abnormal distribution of various myelin gene products^50^ and hypomyelination with subsequent demyelination^51^.

Notably, the *Tubb4a* null mutation had no overt effect on myelination, indicating compensatory functions by other tubulins and a dominant-negative effect of the *taiep* mutation. Nonetheless, the oligodendrocyte-specific expression of this tubulin isoform in the CNS^52^ suggests that its known function as a tubulin stabilizer^53^ is important for myelinating oligodendrocytes.

A growing body of evidence has shown that neuronal electrical activity can stimulate developmental myelination (reviewed in ref. ^54^). Less is known about axon–myelin signaling in the mature fiber. Our finding that the mobility of peroxisomes in myelin’s cytoplasmic spaces is higher in electrically active networks, which, being a rapid response, suggests it involves direct axon–myelin signaling. While mechanistic studies await the analysis of corresponding mouse mutants, we note that mature oligodendrocytes respond to axonal spiking, at least in part through an NMDA receptor-mediated calcium rise in the myelin sheath^40^ that also activates the intracellular transport of the glucose transporter GLUT1 in oligodendrocytes^54^.

We and others already hypothesized a ‘continuity’ of noncompacted myelin as a cytosol-filled rim that flanks compacted myelin. Here, we provide the experimental evidence that this system serves as an active transport pathway long after developmental myelination has been completed; utilizing motor proteins and tubular tracts and exemplified by peroxisome movement. While this description of ‘myelin transport’ (in analogy to ‘axonal transport’) is necessarily incomplete, it already helps to explain aspects of the obscure secondary axonal degeneration of myelin-specific disorders. Adult CNP-deficient mice, modeling a rare human neurological disease^55^ lack integrity of myelin’s cytoplasmic spaces, as CNP normally prevents (MBP-dependent) myelin hyper-compaction and local ‘channel collapse’^5^. Consequently, nuclear-encoded MCT1 probably fails in motor-driven transport via myelin’s cytoplasmic spaces to replenish supplies at the inner tongue of myelin, as suggested by western blotting of a myelin-enriched tissue fraction. Loss of MCT1 in turn predicts poor metabolic coupling with axons^3^, progressive axonal degeneration and the previously described premature death of the animal^23^.

Clinically more relevant are myelin diseases such as MS, where immune-mediated attack on the myelin sheath causes progressive axon loss. We recently found that in human MS and its mouse models, a major contributor to secondary axonal degeneration is myelin injury^56^. We hypothesize this inflammation-mediated myelin injury has the same effect on MCT1 turnover as CNP deficiency, explaining the delayed axonal degeneration occurring months after the primary lesion^56^.

The known neuroprotective function of oligodendrocytes involves the transfer of materials from the oligodendrocyte to the axon^2,3,57,58^. At the inner tongue of normal-appearing fibers in wild-type mice, we observed multivesicular bodies, as reported previously^15^ and fusion profiles (Extended Data Fig. 1g) that could empty exosomes and soluble content, respectively, into the periaxonal space for uptake by the axon.

The brain’s white matter, which contains densely packed, parallel arrays of myelinated axons, is increasingly vulnerable during aging in humans, and small diameter myelinated fibers are particularly at risk^59,60^. If one assumes axon energy insufficiency is a pathological driver, this seems at first counter intuitive because thin axons are predicted to require less energy than thicker ones^61^. A possible explanation relates to the fact that oligodendrocytes that myelinate thin axons extend many more processes than those that myelinate thick axons (reviewed elsewhere^13^). As oligodendrocytes in the aging brain can develop inclusion-containing bulbous swellings (presumably sites of transport stasis) along their processes^43^, it becomes apparent that thin myelinated fibers might be more susceptible due to a greater risk of transport impairment in oligodendrocytes that extend numerous processes to small diameter axons.

## Limitations of the study

A first limitation is that we cannot directly correlate myelin peroxisomal transport with the firing rate of the same axon. However, the wide range of neuronal firing frequencies, corresponded well to the wide variability in the proportion of myelin peroxisomes that exhibited movement. Notably, myelin peroxisome motility was not completely blocked in the presence of TTX, but never reached the mobility seen in cell cultures with intrinsic neuronal electrical activity. Additionally, the number of organelles quantifiable in the inner tongues of myelin in vivo is small (estimated at ~two per sheath), which limits our ability to quantify changes following electrophysiological stimulation of optic nerve. A second limitation of the study is that we could not directly demonstrate that deleting KIF21B in oligodendrocytes impaired TRAM or microtubule dynamics. Finally, the observed loss in western blots, of MCT1 in a ‘myelin-enriched’ spinal cord lysate, provides no direct evidence that the protein is reduced at the inner lip of the myelin sheath.

## Methods

### Mice

Conditional knockout *Kif21b* transgenic mice and ‘reporter’ mice harboring various combinations of tamoxifen-inducible PLPCreER^T2^ (refs. ^62,63^) the ‘stop-floxed’ Cre reporter gene β-actin-tdTomato^64^ and CNP-PexEOS2 (ref. ^65^), were maintained in the Biological Services Facility at the University of Glasgow, under project license PPL P78DD6. All regulated procedures involving these animals were approved by the University of Glasgow Ethical Review Committee, licensed under the UK Animals (Scientific Procedures) Act of 1986, and conducted according to the ARRIVE guidelines. Mice were housed in standard plastic cages or within individually ventilated cages, with 1–4 littermates, and nesting domes. They were maintained with a 12-h dark–light cycle at 19–23 °C, relative humidity 55% (±10%) and provided food and water ad libitum. *Tubba4* conditional knockout mice were bred and housed in the animal facility of the Max Planck Institute for Multidisciplinary Sciences (MPI-NAT). They had ad libitum access to water and food in standard plastic cages with 1–5 littermates in a temperature-controlled room (approximately 21 °C) under a 12-h dark–light cycle. Animal experiments were performed in accordance with the German animal protection law (TierSchG) and approved by the Niedersächsisches Landesamt für Verbraucherschutz und Lebensmittelsicherheit (LAVES) under license 19-42502-04-17/2409, if subject to approval. Procedures were supervised by the animal welfare officer for the MPI-NAT, Göttingen, Germany. The animal facility is registered at the MPI-NAT is registered according to section 11 of the Animal Welfare Act (TierSchG). CD-1 mice were purchased from Charles River Laboratories and housed at the University of Nottingham with ad libitum access to food and water, temperature 22–23 °C and a 12-h dark–light cycle.

#### Transgenic reporter mice

Male and female pairs, which together harbored one copy of each of three transgenes, were mated/time-mated to produce embryos/offspring harboring combinations of transgenes encoding tamoxifen-inducible PLPCreER^T2^ (refs. ^62,63^), the ‘stop-floxed’ Cre reporter gene β-actin-tdTomato^64^ and Cnp-mEos2-PTS1 (ref. ^65^). Approximately one in eight embryos/offspring harbored all three transgenes leading to the expression of EOS2^+^ peroxisomes in tdTomato^+^ oligodendrocytes.

#### *Kif21b* cKO mice

By intercrossing *Kif21b*^*lacZ/neo*^ mutants with mice expressing FLIP recombinase (129S4/SvJaeSor-*Gt(ROSA)26Sor*^*tm1(FLP1)Dym*^*/J*; backcrossed into C57BL/6 N for at least ten generations)^66^ the lacZ/neo cassette was excised in vivo, yielding the ‘conditional ready’ heterozygous *flox* allele in mice carrying the *Kif21b*^*tm2a(KOMP)Wtsi*^ allele (also termed *Kif21b*^*fl/+*^). *Tubb4a*^*fl/+*^ mice were subsequently bred to homozygosity. To inactivate expression of *Kif21b* in oligodendrocytes, homozygous mice were crossed with mice expressing *cre* under the control of the *Cnp* promotor (*Cnp*^*tm1(cre)Kan*^)^23^ to generate *Cnp*^+/*cre*^::*Kif21b*^*fl/fl*^; *Cnp*^+/*cre*^::*Kif21b*^+/+^ or *Cnp*^+/*cre*^::*Kif21b*^+/+^; or *Cnp*^+/+^::*Kif21b*^*fl/fl*^ offspring.

#### *Tubb4a* conditional knockout mice

Embryonic stem cell clones HEPDD0853_6_E02 and HEPD0853_6_B04 harboring the β-tubulin IVA *Tubb4a*^*tm1a(EUCOMM)Hmgu*^ ‘knockout-first’ allele (reporter-tagged with conditional potential) of the *Tubb4a* gene with exons 2 and 3 flanked by *loxP* sites were obtained from the European Mouse Mutant Cell Repository (EuMMCR,) and microinjected into blastocysts by standard procedures. Highly chimeric mice were crossed to C57BL/6N females yielding *Tubb4a*^*tm1a(EUCOMM)Hmgu*^ mutant mice (also termed *Tubb4a*^*lacZ/neo*^*)*. By intercrossing *Tubb4a*^*lacZ/neo*^ mutants with mice expressing FLIP recombinase (129S4/SvJaeSor-*Gt(ROSA)26Sor*^*tm1(FLP1)Dym*^*/J*; backcrossed into C57BL/6N for at least ten generations)^66^ the lacZ/neo cassette was excised in vivo, yielding the ‘conditional ready’ heterozygous *flox* allele in mice carrying the *Tubb4a*^*tm1c(EUCOMM)Wtsi*^ allele (also termed *Tubb4a*^*fl/+*^). *Tubb4a*^*fl/+*^ mice were subsequently bred to homozygosity. To inactivate expression of *Tubb4a* in oligodendrocytes, the loxP flanked exons 2 and 3 were excised in vivo upon the generation and appropriate intercrossing of *Tubb4a*^*fl//fl*^ mice with phenotypically normal *Tubb4a*^*+/+::*^*Cnp*^*cre/+*^ mice that expressed *cre* under the control of the *Cnp* promotor (*Cnp*^*tm1(cre)Kan*^)^23^.

#### Optic nerve electrophysiology

Male CD-1 mice (weight 28–35 g, corresponding to 30–45 days of age) were purchased from Charles River Laboratories and housed at the University of Nottingham with ad libitum access to food and water and temperature 22–23 °C, on a 12-h dark–light cycle. Experiments were approved by the University of Nottingham Animal Care and Ethics Committee and carried out in accordance with the Animals (Scientific Procedures) Act 1986 under appropriate authority of establishment, project and personal licenses.

### Genotyping

Routine genotyping was performed by genomic PCR on ear clip DNA. Ear notches were each lysed in 100 µl 50 mM NaOH at 95 °C for 90 min, then neutralized with 10 µl 1 M Tris buffer (pH 5) followed by thorough vortexing.

*Tubb4a*^*+*^ and *Tubb4a*^*fl*^ alleles were detected using sense primer P1 (5′-GACAGTCAGCAACTGAAGAAGTGGC-3′) together with antisense primer P2 (5′-CTGTTGTCAATCTTGGCCATGAGCC-3′) and P3 (5′-TGAACTGATGGCGAGCTCAGACC-3′) amplifying a 546-bp product for the WT allele, a 748-bp product from the *flox* allele and a 674-bp product from the recombined *flox* allele.

*Cnp*^*cre*^ and wild-type *Cnp1* alleles were identified by genomic PCR with primers P4 (5′-GCC TTCAAACTGTCCATCTC-3′), P5 (5′-CCCAGCCCTTTTATTACCAC-3′), and P6 (5′-CATAGCCTGAAGAACGAGA-3′) amplifying a 700-bp product from the wt allele and a 400-bp product from the Cre or with *Cnp* sense 5′-GCCTTCAAACTGTCCATCTC-3′, Cnp1N2 5′-GATGGGGCTTACTCTTGC-3′ and Puro3 5′-CATAGCCTGAAGAACGAGA-3′, amplifying a 1,180-bp and 894-bp products for wild-type *Cnp* and *Cnp*^*cre*^, respectively.

Mice expressing tamoxifen-inducible tdTomato fluorescent protein and mEos2-PTS-lablelled oligodendrocyte peroxisomes were genotyped using the following primers:

Tom Fw 5′-TACGGCATGGACGAGCTGTACAAGTAA-3′ and Tom Rv 5′-CAGGCGAGCAGCCAAGGAAA-3′, yielding a ~500-bp product.

Eos Fw 5′-CTTCTTACACAGGCCACCATGAGTGCG-3′ and Eos Rv 5′-GGATCCTTACTTAGTTAAAGCTTGGATCGT-3′, yielding an ~800-bp product.

*Plp1* Fw 5′-TGGACAGCTGGGACAAAGTAAGC-3′ and *Plp1* Rv 5′-CGTTGCATCGACCGGTAATGCAGGC-3′ yielding a ~250-bp product.

*Kif21b*^+^ and *Kif21b*^*fl*^ alleles were identified by genomic PCR with primers Fw Kif 83 5′-GGACATGATGGTGATTTAGAGG-3′ and Rv Kif 84 5′-AAACCTGGGCAAAGGCATAC-3′ yielding 825-bp (WT) and 970-bp (*flox* allele) products.

### Cranial window surgery for in vivo peroxisome motility imaging

Cranial windows were prepared as previously described^67^. Transgenic reporter mice (both sexes) (as described above: PLPCreER^T2^; tdTomato; Cnp-mEos2-PTS1) between 10 and 15 months of age were placed under isoflurane anesthesia (induction 5%; maintenance: 1–2%, mixed with 0.6 l min^−1^ O_2_) and kept at 37 °C body temperature with a thermostat-controlled heating plate. The skin over the right cerebral hemisphere was removed, the skull was cleaned, and a 2 × 2-mm region of skull centered over the forelimb region of the primary motor cortex (0–2 mm anterior to bregma and 0.5–2.5 mm lateral) was opened using a high-speed dental drill. A piece of cover glass (VWR, no. 1) was then placed in the craniotomy and sealed with Vetbond (3M) followed by dental cement (C&B Metabond). Carprofen (5 mg kg^−1^) was administered subcutaneously before awakening. To stabilize the head during imaging, a custom metal plate with a central hole was attached to the skull.

### Treadmill training

At 6–8 weeks after cranial window surgery, mice were habituated to the treadmill and head-fixation for increasing periods of time over 5 days (day 1, 5 min head-fixation free exploration of treadmill; day 2, 5 min head-fixation; day 3, 15 min head-fixation; day 4, 30 min head-fixation; and day 5, 45–60 min of head-fixation). Between days 6 and 8, head-fixed mice were imaged during voluntary movement on the treadmill. After training, mice were imaged to identify motile peroxisomes during movement and under anesthesia. During imaging sessions, mice were head-fixed by attaching the head plate to a custom stage and anesthetized with isoflurane when imaging under anesthesia.

### Two-photon live imaging

Images were collected using a Zeiss LSM 7MP microscope equipped with a BiG GaAsP detector using a mode locked Ti:sapphire laser (Coherent Ultra) tuned to 920 nm. The average power at the sample during imaging was 5–30 mW. Vascular and cellular landmarks were used to identify the same cortical region when switching between voluntary movement and anesthesia. Image stacks were acquired using a Zeiss W plan-apochromat ×20/1.0 NA water immersion objective giving a volume of 425 × 42 × 114 μm (2,048 × 2,048 pixels; corresponding to layer I, 0–114 μm from the meninges) from the cortical surface. Image timelapses were acquired over 20 min, with a frame acquired from two *z*-planes every 10 s, giving a total volume of 425 × 425 × 3 μm (1,024 × 1,024 pixels). Image stacks were taken before and after timelapses, first during voluntary movement on the treadmill, then under anesthesia.

### Quantification of in vivo peroxisome motility

Images were analyzed using ImageJ (v.1.54f) unless otherwise specified. Image timelapses were registered using the TurboReg plugin. Image stacks taken before and after timelapses were registered with Poorman 3Dreg plugin for X/Y registration (ImageJ v.1.46r) followed by Correct3D drift plugin (tdTomato channel, rigid body registration, 30 × 30 × 20 pixels *x*/*y*/*z*; ImageJ v.1.54 f). Analysis of motile peroxisomes was done by comparing image stacks before and after timelapses, allowing 30 min between analysis time points. Analyzed images were pre-processed with a median filter (radius of one pixel) to denoise and aid identification of individual myelin sheaths and peroxisomes. Peroxisomes were considered ‘motile’ if they were present in the image stack before the timelapse, but not in the image stack after the timelapse, or if they were not present pre-timelapse and appeared in the post-timelapse image stack. Stable peroxisomes were present in the same position in both image stacks. Only peroxisomes within myelin sheaths were quantified. Sheaths were identified as previously described^68,69^ sheaths with at least one paranode and with an identifiable process connecting to the cell soma were quantified. Between 19 and 31 myelin sheaths were analyzed in each of five oligodendrocytes per mouse. For presentations in figures and videos, images were processed with a one-pixel median filter and gaussian blur and brightness and contrast levels were adjusted for clarity. Due to the possibility of double counting single motile peroxisomes more frequently in the treadmill condition, we recalculated the data taking account of the maximum possible number of double counts; this did not change the conclusion of the experiment presented in Fig. 3h.

### Transmission electron microscopy of conventionally fixed tissue

Transcardiac perfusion of mice was performed under the effect of a lethal dose of Avertin or Dolethal. For TEM, mice were perfused with 4% paraformaldehyde (PFA), 2.5% glutaraldehyde in 0.1 M phosphate buffer, post-fixed in 4% PFA overnight and subsequently transferred to 1% PFA (conditional *Tubb4a* knockout mice and controls) or with 4% PFA and 5% glutaraldehyde in cacodylate buffer, and post-fixed in the same for 24 h to 3 weeks (*Kif21b* cKO mice and controls). Optic nerves were subsequently dissected and processed for TEM, as previously described^70^. Sampling was performed as depicted in Supplementary Methods.

Following electrophysiology of CD-1 mouse optic nerves, paired nerves (stimulated and nonstimulated) were immersed in 4% PFA and 5% glutaraldehyde in cacodylate buffer for at least 1 week and processed and sectioned as above. Morphometry and quantification of electron micrographs were performed using Fiji^71^.

### Transmission electron microscopy of high-pressure frozen tissue

Tissue was collected and processed as described elsewhere^72^. In brief, mice at 6 or 24 months of age were killed. The optic nerves were dissected, cut and high-pressure frozen using 20% polyvinylpyrrolidone (PVP) as a filler and the HPM100 high-pressure freezer (Leica). An automatic freeze substitution unit (Leica, AFSII) was used to perform the freeze substitution. 0.1% tannic acid in acetone was incubated at −90 °C for 100 h followed by three acetone rinses for 30 min each. Then, 2% OsO_4_, 0.1% uranyl acetate in acetone was used for contrasting the samples (7 h at −90 °C). The temperature was automatically raised to −20 °C within 14 h, kept there for 16 h and raised to 4 °C within 2.5 h. OsO_4_ was removed by washing four times with acetone for 30 min. The nerves were then rinsed with acetone for 1 h at room temperature. The samples were incubated with increasing concentrations of resin (2:1, 1:1 and 1:2) for 2 h each and left in 90% EPON (Serva) overnight. On the next day the samples were incubated with 100% EPON for 4.5 h and polymerized for 24 h at 60 °C.

### Immunoelectron microscopy

Mice at 2 months were fixed by transcardial perfusion with 4% formaldehyde and 0.2% glutaraldehyde in 0.1 M phosphate buffer containing 0.5% NaCl. Dissected spinal cord was sectioned using a vibratome (VT1200S, Leica Microsystems, Wetzlar). Sections were infiltrated in 2.3 M sucrose in 0.1 M phosphate buffer overnight and selected dorsal white matter regions mounted onto aluminum pins for ultramicrotomy and frozen in liquid nitrogen. Ultrathin cryosections were picked up in a 1:1 mixture of 2% methylcellulose and 2.3 M sucrose. For immunolabeling, sections were incubated with anti-catalase (1:100 dilution, rabbit antiserum, Rockland; no. 200-4151, RRID:AB_2071858), which was detected with protein A-gold (10 nm) (Cell Microscopy Core, Department of Cell Biology, UMC Utrecht, The Netherlands). As a second marker, peroxisomes were identified with antibodies to peroxisomal membrane protein PMP70 (1:100 dilution, rabbit polyclonal, Abcam (ab3421, RRID:AB_2219901), which was detected with 15 nm protein A-gold. Sections were analyzed with a LEO EM912 Omega (Zeiss, Oberkochen) and digital micrographs were obtained with an on-axis 2,048 × 2,048-CCD camera (TRS, Moorenweis).

### White matter morphometry

Optic nerve cross-sections taken mid-way between the retina and the extraorbital region of the nerve from mice of both sexes were imaged at ×12,000 on a JEOL 1200 EX transmission electron microscope (*Kif21b*, *Tubba4* cKO mouse optic nerves and nerves the underwent electrophysiology) or a TEM912AB electron microscope (aged mice). Images of optic nerve cross-section were captured randomly at ×12,000 magnification using Olympus I TEM Software (8–12 images per nerve, each ~74 μm^2^ (optic nerve electrophysiology experiment; Extended Data Fig. 1) or 12–20 images per nerve, each ~74 μm^2^ (Figs. 4 and 5 and Extended Data Fig. 5)) or at ×3,150 (five images per nerve, each 480 μm^2^ (aged mice, Fig. 6)) using the ImageSP software (TRS, SysProg, ImageSP, v.1.1.4.62), respectively. Images were opened in Fiji image analysis software^33^, and the relevant feature was counted using the ImageJ Cell Counter plugin within and on west and south borders of an area of interest. The sum of the counts from all micrographs from a single nerve was converted to numbers per mm^2^. Some counts were converted to fibers per nerve after measuring the area of the optic nerve cross-section from toluidine blue stained resin sections at ×10 magnification. For quantifying organelles in inner tongue, organelles (Fig. 1) were identified, and the numbers of organelles was divided by the number of myelinated fibers to determine the number of organelles per fiber cross-section. To determine organelle diameter, the area of each organelle was measured in Fiji^71^ by tracing the organelle bounds and the diameter calculated, assuming the area was circular. Organelles were assumed to be spherical, and the maximum diameter observed on optic nerve cross-sections was assumed to represent the maximum diameter in the longitudinal direction (along the sheath). To calculate the approximate number of organelles per inner tongue we divided the average sheath length (76,000 nm) by tissue section thickness (50 nm) to determine the number of sections per sheath if sectioned serially (*n* = 1,520). Assuming each organelle is ~0.3 μm in diameter, then the organelle would be contained in 300 nm/50 nm = 6 of these 1,520 sections. This represents a 1 in 253 chance of encountering an organelle in any one fiber cross-section.

### Tissue collection for light microscopy (frozen sections)

Mice were perfusion fixed under the effect of a lethal dose of Dolethal, with 0.9% saline followed rapidly by 4% PFA and immersed in 4% PFA for a minimum of 24 h before the optic nerve and spinal cord were dissected. Dissected tissues were transferred into 20% sucrose in PBS until they sank. Tissues were frozen in OCT (Sakura Finitek) in liquid nitrogen-chilled isopentane. Ten µm thick sections were cut on a cryostat (Bright Instruments) and mounted on Colorfrost Plus charged slides (Thermo Fisher Scientific). Tissue sections were permeabilized in methanol for 10 min at −20 °C and immunostained with rabbit anti-CASPR1 (gift from O. Peles) by incubation overnight at 4 °C in blocking buffer (10% goat serum, 1% bovine serum albumin (BSA), 0.1% Tween and 0.9% NaCl in PBS). Bound primary antibodies were detected using Alexa 647 goat anti-rabbit IgG (1:1,000 dilution; Invitrogen).

### Tissue collection for light microscopy (paraffin sections)

Mice were anesthetized with a lethal dose of avertin and perfused with Hanks balanced salt solution followed with 4% PFA in 0.1 M phosphate buffer. The brains were dissected, post-fixed overnight in 4% PFA, paraffinized using a Microm STP 120 Spin Tissue Processor (Thermo Scientific) and subsequently embedded in paraffin blocks on a HistoStar embedding workstation (Epredia).

### Immunohistochemistry and quantification on paraffin sections

The 5-μm brain paraffin sections were cut on a RM2155 (Leica) rotary microtome and mounted on Histobond adhesive microscope slides. Slices were deparaffinized at 60 °C and rehydrated using xylene and a descending ethanol series. For heat-induced epitope retrieval, sections were treated in sodium citrate buffer (0.01 M, pH 6.0) in a 400-Watt microwave for 10 min, followed by a 20-min cooldown period. For immunohistochemical antibody detection using a chromagen, endogenous peroxidase activity was blocked with 3% hydrogen peroxide. Subsequently, sections were blocked for 1 h at room temperature) in 20% goat serum in PBS and incubated with primary antibodies (anti-MAC3, anti-IBA1, anti-APP) at 4 °C overnight. The LSAB2 system-HRP kit (DAKO) was used and the HRP substrate 3,30-diaminobenzidine (DAB) was applied using the DAB Zytomed kit (Zytomed Systems). Nuclei were labeled by Hematoxylin stain. Imaging was performed using a Zeiss AxioImager Z1 bright-field light microscope equipped with a Zeiss AxioCam MRc camera using ZEN 2012 blue edition software. Images of anterior corpus callosum and motor cortex (bregma 0.74 mm) were color deconvoluted using the ‘Color Deconvolution’ (v.1.3) plugin in Fiji/ImageJ. IBA1^+^ and MAC3^+^ areas were quantified using thresholding, and APP spheroids were quantified manually using the Cell counter plugin from Fiji/ImageJ. All data were normalized to the respective quantified areas.

### Single-molecule FISH

Candidate smFISH probe sequences recognizing mouse *Mbp* (NM_001025251) or *Mobp* (NM_001039365) were generated using Stellaris Probe Designer v.4.2 (https://www.biosearchtech.com/stellaris-designer) with the following parameters: organism, mouse; masking level, 5; oligonucleotide length, 18 nt; minimum spacing length, 3 nt. BLAST was used to remove potential off-target oligonucleotide sequences (e-value cutoff of 1 × 10^−2^). The 3′ ends of oligonucleotides were labeled with Alexa Fluor 647^73^ normalized to 25 µM. All probe sets used had a degree of labeling >0.99. Probe sequences are provided in Supplementary Table 2.

smFISH was conducted as previously described^74^ with minor modifications. The 10-µm sections from the adult optic nerve were fixed in 4% PFA for 10 min. Sections were washed twice in PBS and stored in 70% ethanol at 4 ˚ C until use. Tissue was rehydrated in successive incubations (35%, 17.5% and 0%) ethanol 2× SSC, for 5 min each at room temperature. Pre-hybridization, sections were washed in prewarmed wash solution (2× SSC, 10% formamide and 0.1% Tween 20) twice for 20 min each at 37 °C. Hybridization was carried out in 2× SSC, 10% formamide, 10% dextran sulfate and 0.1% Tween 20, containing 250 nM smFISH probes overnight at 37 °C. Sections were then washed in prewarmed wash solution for 20 min and stored in PBS. Before immunocytochemistry, RNASecure (Invitrogen) was added to Odyssey blocking solution (Li-Cor), and applied to cells for 1 h at room temperature. Sections were then incubated in primary antibody solution (anti-CNPase; Abcam) in Li-Cor blocking buffer overnight at 4 °C. Sections were washed in PBS and incubated in Alexa Fluor 568 anti-mouse secondary antibody in Li-Cor blocking buffer (Invitrogen) for 1 h at room temperature. Sections were washed in PBS and incubated in TrueBlack Plus (Biotium) to reduce autofluorescence, according to the manufacturer’s instructions, and were then rinsed and mounted with Mowiol with DAPI.

Sections were imaged using an Olympus SpinSR (CSU-W1) spinning disk confocal microscope with a Prime BSI sCMOS camera ×100 oil (1.45 NA, UPLXAPO100XO). Image voxel sizes were 0.067 × 0.067 × 0.2 µm (*x*:*y*:*z*). The photoconvertible fluorescent protein mEos2-PTS1 exhibits bleed through into the 550–600 nm filter range, overlapping with the tdTomato fluorescence emission spectrum. To correct for this, the tdTomato fluorescence channel was post-processed using the following method. mEos2-PTS1 and tdTomato images were acquired separately with 488 nm (green channel) and 561 nm (red channel) presets using an Olympus SpinSR10 microscope. The green channel image stack underwent background subtraction (radius of 10 px), followed by the addition of a uniform pseudo-background intensity of 15 to prevent division by zero. This value was derived from the typical median intensity of a Gaussian-filtered (σ = 10) green channel image. The red channel image stack was similarly background-subtracted (radius of 50 px) and then divided by the processed green channel image. The resulting 32-bit float image was converted to 16-bit integer values using the min–max normalization method.

### Quantitative RT–PCR

RNA was extracted from brain lysates and purified myelin-enriched fractions using the miRNeasy Mini kit (QIAGEN) and the concentration was increased using Co-precipitant pink (Bioline), if necessary. The purity and concentration of RNA was evaluated using a NanoDrop spectrophotometer and RNA Nano (Agilent). Complementary DNA was synthesized using Superscript III (Invitrogen) according to the manufacturers’ instructions. Quantitative PCR with reverse transcription (qRT–PCR) was performed in triplicate with the GoTaq qPCR Master Mix (Promega) on a LightCycler 480 Instrument (Roche Diagnostics). Expression values were normalized to the mean of the housekeeper genes *Atp1a1*, *Hprt* and *Rplp0*. Relative expression changes were analyzed using the 2ΔΔ*C*_T_ method, normalized to mean expression in P20 wild-type myelin-enriched fractions (set to 1). All primers were designed with Universal Probe Library from Roche Applied Systems (https://www.roche-applied-science.com) and validated with National Institutes of Health PrimerBlast. All primers were intron-spanning and were specific for *Slc16a1* (forward 5′-GGATATCATCTATAATGTTGGCTGTC-3′, reverse 5′-GCTGCCGTATTTATTCACCAA-3′; *Atp1a1* (forward 5′-GGCCTTGGAGAACGTGTG-3′, reverse 5′-TCGGGAAACTGTTCGTCAG-3′); *Hpr*t (forward 5′-CCTGGTTCATCATCGCTAATC-3′, reverse 5′-TCCTCCTCAGACCGCTTTT-3′) and *Rplp0* (forward 5′-GATGCCCAGGGAAGACAG-3′, reverse 5′-ACAATGAAGCATTTTGGATAATCA-3′).

### Tissue collection and western blotting of myelin-enriched fraction and optic nerves

Male C57BL6/N wild-type mice and CNP null mutant mice (*Cnp*^*tm1(cre)Kan*^; ref. ^23^) from breeding colonies at the Max Planck Institute of Multidisciplinary Sciences and 12 male and 8 female *Cnp*^+/+^::*Kif21b*^*fl*/*fl*^, *Cnp*^+/*cre*^::*Kif21b*^+/+^ or *Kif21b* ^+/*fl*^ and *Cnp*^+/*cre*^::*Kif21b*^*fl/fl*^ from breeding colonies at the University of Glasgow were used. A lightweight membrane fraction enriched for myelin was purified from mouse brain hemispheres or spinal cords, as described elsewhere^35^). Optic nerves were homogenized in ROLB Buffer, pH 7.4 (10 mM HEPES, 50 mM NaF, 25 mM Na_4_P_2_O_7_, 80 mM KH_2_PO_4_, 5 mM EDTA and 1% SDS with protease inhibitor) using a plastic pestle and mortar then centrifuged at 16,000*g* for 10 min, 4 °C and the soluble fraction was aspirated. Protein concentration was determined using the DC protein assay (BioRad) or a BCA Protein Assay kit (Thermo Fisher) according to the respective manufacturer’s instructions. For immunoblotting of brain ‘myelin’ spinal cord ‘myelin’ and optic nerve, samples were resolved on 4–12% Bis-Tris gel at 200 V and transferred onto PVDF membrane using either semi-dry method (Trans-blot; BioRad) or a wet gel transfer (Mini Trans-blot; BioRad). Semi-dry transfers were run at 1.3 mA cm^−2^ for 1 h ± 15 min and wet gel transfers were run overnight at 35 V or for 1 h at 100 V. Membranes were blocked with 5% milk in Tris-buffered saline with 0.01% Tween 20 (TBS-T) then incubated in primary antibody in blocking buffer overnight at 4 °C. Primary antibodies were specific for SCL16a1/MCT1 (1:50 to 1:1,000 dilution (refs. ^75,76^), ATP1a1 (1:2,000 dilution; Abcam) KIF21B (1:50 dilution; HPA027274, Sigma; adsorbed against *Kif21b* KO brain tissue), CNP (1:2,000 dilution; ab6319, Abcam), MOG (1:2,000 dilution; gift from C. Linington) and β-actin (1:5,000 dilution; A1978, Sigma). Membranes were thoroughly washed (1× TBS/T) and binding was detected using an appropriate peroxidase-conjugated secondary antibody (anti-mouse/rabbit IgG HRP-linked, Cell Signaling Technologies). Washing was repeated then the sample was incubated for 1 min in WesternBright ECL mix (K-12045-D20, Advansta). Immunoblots were scanned using the Intas ChemiCam system or using the Li-Cor C-DiGit blot scanner.

### Myelinating cell cultures

Pregnant ‘reporter’ mice were killed on embryonic day 13 (E13; day of plug being E0) by cervical dislocation followed by exsanguination or CO_2_ exposure followed by cervical dislocation. Following removal of the uterine horns, isolation of the embryos and spinal cord removal, all embryonic cords were pooled and used to generate a single cell suspension for culture, as described elsewhere^77^ resulting in a population of cells with a mixed genotype. Myelinating cultures were maintained for up to 50 days in vitro (DIV), in a cell culture incubator at 37^o^C in humidified 5% CO_2_ in serum free differentiation medium (DM) as described^77^. One micromolar (final) 4-hydroxy tamoxifen was administered for 1 h on DIV 21 ± 3 days to induce tdTomato expression in a subpopulation of oligodendrocytes.

### Neuronal cell cultures

These were established as for myelinating cell cultures, except that the dissociated spinal cord cells were plated in 80 µl serum-containing plating medium (50% DMEM, 25% HBSS and 25% horse serum) at 80,0000 cells per well in Seahorse XFp Miniplates (Agilent, 103025-100). After 2 h, the serum-containing media was removed and replaced with 125 µl neurobasal medium (Gibco, A2477501) supplemented with B-27 (Gibco, 17504044), 10 µM 5-fluoro-2′-deoxyuridine (Sigma-Aldrich, 343333), 10 ng nerve growth factor (Sigma-Aldrich, N6009), 10 mM glucose (Agilent, 103577-100), 0.5 mM glutamine (Agilent, 103579-100) and 0.227 mM pyruvate (Agilent, 103578-100). The cells were maintained until DIV 15 ± 1 day and the medium was changed every 2 days.

### Seahorse neuromodulator assay on neuronal culture

The cartridges for the Seahorse XF Bioanalyser (Agilent) were prepared according to the manufacturer’s instructions. On the day of the assay, neurobasal medium was changed to 180 µl of the Seahorse assay medium (Agilent, 103680-100, supplemented with 2 mM glutamine, 1 mM pyruvate and 10 mM glucose) and equilibrated in a non-CO_2_ incubator for 1 h at 37 °C. The assay was three cycles of baseline measurements followed by injection of 100 μM PTX and two measurement cycles, followed by injection of 1 μM TTX and two measurements cycles. The total duration for the assay was 40 min and OCR and extracellular acidification rate were recorded, with values for each phase averaged and plotted over time.

### Treatment of myelinating cultures with nocodazole

On DIV 27 ± 3 days, myelinating cultures were treated for 3 h at 37 °C in a humidified 5% CO_2_ incubator with nocodazole (final concentration 20 μM; 1.2 μl of 5 mg ml^−1^ in dimethylsulfoxide (DMSO) was added per 1 ml medium; SML1665; Sigma-Aldrich).

### Treatment of cultures with neuromodulators

On DIV 29 ± 5 days, myelinating cultures were treated for ≥5 min with either PTX (final concentration 100 μM; 1 μl stock solution in DMSO in 1 ml medium; P1675; Sigma-Aldrich) or TTX (final concentration 1 µM; 1 µl stock solution in water in 1 ml medium; 1078; Tocris).

### Live imaging of cell cultures

For live imaging, myelinating cultures were plated on custom-made imaging dishes, prepared from 35-mm diameter Petri dishes (353001; Falcon), with three 8-mm diameter circular holes in the base and a 25 mm diameter glass coverslip (630-2213; VWR) glued underneath^77^. Live tdTomato-rendered oligodendrocytes with mEos2-PTS-lablled peroxisomes were imaged using an inverted Zeiss Observer Z1 Spinning Disc confocal microscope, equipped with a Yokogawa CSU-X1 filter wheel and spinning-disc unit, a Photometrics Evolve 512 delta TEM-CCD camera, with 488 nm (300–600 ms exposure, 4–9% power) and 568 nm (200–400 ms exposure, 4–6% power) laser lines and a ×63/1.4 Oil Pln Apo objective and Zen software (2012, Blue Edition). Alternatively, an inverted Leica DMi8 widefield microscope, with a Hamamatsu ORCA-Flash4.0 v.3 Digital camera C13440-20C, HC PL APO CS2 ×63/1.40 Oil objective, and LAS-X software (v.3.7.1) or an inverted Zeiss Observer Z1 epi-fluorescence microscope equipped with an AxioCam MRc3, ×0.63 Camera Adaptor, ZEN 2012 blue edition software using a Zeiss plan-apochromat ×63 1.4 oil objective, were used. Cells were maintained at 37 °C on a heated stage insert and gassed with humidified 5% CO_2_ to maintain physiological pH, which was monitored on the basis of the color of the phenol red-containing cell culture medium. To record peroxisome movement, frames were captured every 10 s over a 10-min period, in the 568 (tdTomato) and 488 (mEos2-PTS) channels.

### Quantification of peroxisome motility in vitro

The numbers of peroxisomes in oligodendrocyte processes or sheaths were counted manually using the multipoint tool in Fiji^71^. To calculate the percentage of motile peroxisomes in oligodendrocyte processes or sheaths, the total number of peroxisomes per sheath/process and the number of motile peroxisomes were recorded. Peroxisomes that moved ≥3 µm were considered ‘motile’. To measure the distances traveled, displacement and velocity of motile peroxisomes we performed manual tracking using the MTrackJ plugin in Fiji. Velocity was calculated by dividing the total distance traveled by the number of frames in which the peroxisome was motile multiplied by 10 s (the interval between frames). In MTackJ, displacement represents the net change in position of a tracked object from its starting point to its final point, measured as the length of a straight line connecting them.

For motile peroxisomes in processes (between the soma and the sheaths), the direction of movement in relation to the cell body was also recorded, and the net distance traveled in each direction (anterograde or retrograde) was quantified. First, the length of each process was measured using MTrackJ. Second, as process lengths varied, and hence, impacted distance available, the distance traveled by each peroxisome (measured as above) was normalized to distance per 100-µm process. If there were no motile peroxisomes in a process, zero was recorded for both anterograde and retrograde movement. If movement was in only one direction, zero was recorded for the opposite direction. For example, in a process in which one peroxisome moved 50 μm per 100-μm process length in the anterograde direction, the distances were defined as anterograde 50 μm, retrograde 0 μm.

### Culture of glial cells on glass or on nanofibers

Spinal cords were dissected from P2–5 ‘reporter’ mice, digested using trypsin (0.125%) in HBSS minus Ca^2+^ and Mg^2+^, and dissociated into a single cell suspension by trituration through 21 and 23G needles. Only glial cells survive this process, eliminating almost all neurons from the cell culture. Cells were plated at 50,000 cells per well on 4-μm diameter poly-L-lactic acid electrospun fibers in 12-well cell crown scaffolds (The Electrospinning Company^78^) or at 30,000 cells per 11-mm diameter glass-bottomed well in serum-containing plating medium (see section on ‘Myelinating cultures’). The following day, the plating medium was removed and the glial cells were maintained in Sato’s medium (DMEM, glucose (25 mM), pyruvate (1 mM), penicillin–streptomycin (1% v/v), apo-transferrin (5 μg ml^−1^), sodium selenite (5 ng ml^−1^), insulin (5 μg ml^−1^), putrescine (16 μg ml^−1^), progesterone (7.3 ng ml^−1^), T3 (0.5 nM) and horse serum (1% v/v); Fig. 3c) or in DM, as for myelinating cell cultures (Extended Data Fig. 4 and Supplementary Video 9) with the medium replenished 50:50 every 7 days.

For live-imaging peroxisomes on myelinated nanofibers, the nanofiber crown was carefully removed from the 12-well plate and placed in a glass-bottomed imaging dish with a small volume of medium. The nanofibers were carefully cut at the point of attachment to the crown, so that they collapsed onto the glass for imaging using a Zeiss inverted spinning-disc confocal microscope as for other live imaging.

### Immunocytochemistry

Cell cultures were fixed in 4% PFA at room temperature for 10 min, or by the addition of 8% PFA (37 °C) to cell culture medium (1:1 v:v), then permeabilized for 10 min in either 0.5% Triton X at room temperature or in absolute methanol at −20 °C. Following washes in PBS, blocking buffer (10% goat serum, 1% BSA, 0.1% Tween and 0.45 g NaCl in 50 ml PBS) was applied for 1 h at room temperature and then cells were incubated overnight in primary antibody at 4 °C, in 10% goat serum in PBS or blocking buffer. After thorough washing, secondary antibodies were applied at 1 in 1,000 for ~1 h at room temperature. Finally, cells were washed in PBS and rinsed in water, then coverslips were mounted on glass slides in Mowiol containing DAPI (2 µg ml^−1^; Invitrogen). For immunocytochemistry of microtubules, cells were first incubated for 1 min with prewarmed extraction buffer (0.3% Triton X and 0.1% glutaraldehyde in BRB80 buffer (BRB80 buffer: 80 mM PIPES, 1 mM EGTA and 4 mM MgCl_2_, pH 6.8)^31^ then fixed for 10 min in 4% PFA and permeabilized for 10 min in 0.25% Triton X before blocking for 1 h in 3% BSA/PBS at room temperature. Cells were next incubated for 1 h with primary antibody at room temperautre, diluted in 3% BSA/PBS, washed in PBS, then incubated for 1 h at room temperature with secondary antibodies diluted in 3% BSA/PBS. Coverslips were washed and mounted as described above. Primary antibodies were; mouse IgG2a anti-β-tubulin III (1:200 dilution; T8578; Sigma), mouse IgG1 anti-β-tubulin IV (1:400 dilution; ab11315; Abcam), rat anti-MBP (1:800 dilution; ab7349; Abcam), mouse IgG1 anti-CNP (1:800 dilution; ab6319; Abcam), rabbit anti-CASPR1 (1:1,000 dilution; provided by E. Peles), mouse IgG2a anti-Ermin (1:500 dilution; MABN323; Merck); mouse IgG3 anti-tyrosinated tubulin (1:300 dilution; T9028; Merck); mouse IgG2b anti-acetylated tubulin (1:300 dilution; T7451; Merck); rabbit anti-detyrosinated tubulin (1:300 dilution; ab48389; Abcam); rabbit anti-PEX14 (1:750 dilution; ab183885; Abcam). Bound primary antibodies were detected using Alexa 488, 568 or 647 goat anti-mouse IgG, IgG1, IgG2a, goat anti-rabbit IgG or goat anti-rat IgG (1:1,000 dilution; Invitrogen).

To stain cells cultured on nanofibers, the nanofiber crown was transferred between wells of a 12-well dish for permeabilization of cells, antibody incubations and washing, as above. The nanofiber crowns were placed on glass slides and the fibers were sandwiched between the slide and a 13-mm glass coverslip, using Mowiol with DAPI. The crown was removed 2 days later, after the Mowiol had hardened, as described^79^.

### Transfection with sensors of microtubule post-translational modifications

Myelinating cultures were incubated with DharmaFECT 1 transfectant reagent (T-2001-01; Horizon) according to manufacturer’s instructions and with 1.125 µg ml^−1^ tyrosinated tubulin sensor TagRFP-T AlaY1 (ref. ^29^) (158751; Addgene) or Stable Microtubule-Associated Rigor-Kinesin (StableMARK)^30^ for 9 h. Cells were immediately fixed in 4% PFA/PBS and immunostained as described in the ‘Immunocytochemistry’ section.

### Fluorescence microscopy

Fixed cell cultures and tissue sections were generally imaged with an inverted Zeiss Observer Z1 epifluorescence microscope equipped with an AxioCam MRc3, ×0.63 Camera Adaptor, ZEN 2012 blue edition software using Zeiss plan-apochromat ×63 1.4 oil objective. High-resolution images were taken using a Zeiss LSM 880 confocal microscope or Zeiss Z.1 AxioImager with ApoTome attachment, using Zen software and Plan-Neofluar ×40/1.30 oil objective or Plan-Neofluar ×63/1.30 oil objectives. Cells on nanofibers were imaged on an Olympus IX70 microscope, QIcam Fast digital camera (QImaging Surrey) and Image-Pro Plus 6 software (Media Cybernetics).

### Quantifying peroxisomes in paranodes

Myelinating cultures were treated with neuromodulators, TTX or PTX (see ‘Treatment of cultures with neuromodulators’ section) for 5 min, 2 h or 24 h, then immediately fixed in 4% PFA/PBS and either immunostained with anti-CNP (see ‘Immunocytochemistry’ section) and mounted on glass slides in Mowiol or mounted in Mowiol without immunostaining when expressing tdTomato. Coverslips were scanned in the green (mEos2-PTS-labeled peroxisomes) and red (oligodendrocytes stained with anti-CNP or labeled with tdTomato) channels, and oligodendrocytes with mEos2-PTS-peroxisomes in the soma were selected at random. A *z*-stack (0.32-μm steps) was captured of paranodal regions using a Zeiss Observer Z1 epifluorescence microscope. Numbers of peroxisomes in each paranode were counted manually in Fiji or in Zen 2012 (Blue edition) in a minimum of ten paranodes per treatment, per independent experiment.

### Electrophysiology of optic nerve

Following decapitation, the optic nerves were cut behind the eye orbit then exposed by lifting the cerebral hemispheres rostro-caudally. They were cut at optic chiasm (5–7 mm length) and gently freed from their dural sheaths then placed in an interface perfusion chamber (Medical Systems), as described^79^. Optic nerves were maintained at 37 °C using a TC-202A bipolar temperature controller (Digitimer) and perfused with artificial cerebrospinal fluid (aCSF) that contained 153 mM Na^+^, 3 mM K^+^, 2 mM Mg^2+^, 2 mM Ca^2+^, 143 mM Cl^−^, 26 mM HCO_3_, 1.25 mM HPO_4_^2−^ and 10 mM glucose. The aCSF was bubbled with a gas mixture (95% O_2_:5% CO_2_) to maintain pH at 7.45. Tissue was oxygenated by a humidified gas mixture (95% O_2_:5% CO_2_) that flowed over its surface. Nerves were allowed to equilibrate for 30 min then a stimulating suction electrode backfilled with aCSF was attached to the proximal end of one nerve. A recording electrode filled with aCSF was attached to the distal end of the same nerve to record the compound action potential (CAP), which was evoked by a 125% supramaximal stimulus (30 µs in duration: a Grass S88 stimulator connected to two SIU5 isolation units in parallel capable of delivering two independently controllable pulses). Nerves were stimulated at 7 or 50 Hz for 20 min. CAPs were recorded from a suction electrode attached to the distal end of the nerve connected to an SR560 low-noise preamplifier (Stanford Research Systems): the signal was amplified up to 1,000× and filtered at 30 kHz. The differential output was fed into a HumBug Noise Eliminator (Digitimer) to remove mains frequency noise (50 Hz). The signal was acquired via an Axon Digidata 1550B using Clampex 11.3 (Molecular Devices). The control nerve remained untouched on the perfusion chamber, and when the stimulation protocol was completed, both nerves were transferred rapidly to fixative as described in ‘Tissue collection and fixation’. The number of fibers examined per optic nerve varied from 202 minimum to 414 maximum.

### Electrophysiology of myelinating cultures

Myelinating cultures were transported between sites at room temperature in a sealed box containing humidified 5% CO_2_ (maximum journey time 1.5 h) and allowed to equilibrate at least 1 h in cell culture incubator (37 °C, 5% CO_2_). One glass coverslip at a time was removed from the cell culture medium dish and placed in a clean 35-mm diameter Petri dish containing prewarmed extracellular solution, then viewed using an Olympus IX71 inverted microscope with epifluorescence. Whole-cell voltage and current clamp recordings were performed on DIV 22–29 cultures using an Axopatch 200B amplifier with a National Instruments NIDAQ-MX digital acquisition system and results acquired and analyzed with WinEDR v.3.9.1 software. Experiments were performed at 35–37 °C in atmospheric air using an extracellular solution containing the following ion concentrations to mimic the potassium concentration of the cell culture media and concentration of other ions in cerebrospinal fluid 144 mM NaCl, 5.3 mM KCl, 2.5 mM CaCl_2_, 1 mM MgCl_2_, 1 mM NaH_2_PO_4_, 10 mM HEPES and 10 mM glucose (pH 7.4)^80^. Temperature was controlled using a TC-324C automatic temperature controller (Warner Instruments). The pipette solution contained 130 mM K-gluconate, 4 mM NaCl, 0.5 mM CaCl_2_, 10 mM HEPES and 0.5 mM EGTA, (pH 7.2) with KOH. The osmolarity of the external solution was 312 mOsm and the internal solution was 270 mOsm, both adjusted with sucrose. Borosilicate glass pipettes were pulled to a resistance of 5–20 MΩ.

To record from neurons, electrodes were brought into contact with the cell membrane and gigaOhm seals formed by gentle suction supplied from a syringe and water filled U tube. Firm suction was then used to break the membrane under the pipette to form a whole-cell recording. Recordings exhibiting a leak of more than 100 pA were rejected at this stage. Within 2 s of whole-cell access, the amplifier was switched to current clamp mode to enable instantaneous membrane potential to be estimated. Recordings were made for 2 min in current clamp mode to measure neuronal activity at baseline and after 5 min treatment with neuromodulators.

Action potential firing frequency was calculated using the frequency analysis function in WinEDR (Strathclyde electrophysiology software, http://spider.science.strath.ac.uk/sipbs/software_ses.htm). Data were presented as the number of action potentials per second (Hz). Action potentials were defined as regenerative, all-or-nothing changes in membrane potential that produced an overshoot beyond 0 mV.

### Statistical analysis

Data were analyzed using GraphPad Prism v.10, with significance set at *P* ≤ 0.05. Statistical tests are indicated in the relevant figure legends.

### Statistics and reproducibility

Investigators were blinded to experimental conditions for data analysis of peroxisome tracking (in vivo and in vitro) and white matter morphometry. Further, electron micrographs were captured as described in Supplementary Methods. Investigators were not blinded for electrophysiology (experimenter added the drug) and western blot analysis, where the samples were loaded on gels in a predetermined order, for example WT, Het control and cKO. For peroxisome tracking, cells were excluded from analysis if there were <5 peroxisomes per sheath/process observable in the timelapse image. For morphometry, samples were (partially) excluded if the fixation was not adequate for identifying myelin organelles. One *Kif21b* cKO optic nerve was excluded from morphometry because it seemed shrunken and abnormal. Cell cultures were excluded from analysis if they did not meet quality control criteria (where tdTomato-rendered myelin did not seem smooth, instead appearing ‘blebbed’). Cell culture dishes were randomized for treatment with nocodazole or neuromodulators. *Kif21b* cKO mice and controls were generated by breeding *Cnp*^*cre*/+^::*Kif21b*^*fl/*+^ × *Cnp*^+/+^::*Kif21b*^*fl*/+^ so all offspring of the relevant genotypes, whether male or female, were used for morphometry and western blotting. Sample size was between 3 and 9 independent experiments (i.e. 3–9 controls and 3–9 matched experimental samples), except in two cases, where only two matched pairs were analysed (Fig. 5a (anti-MOG on 1 year samples) and Fig. 5d (inner tongue organelles at P40 and P120). No statistical tests were undertaken on these. The NC3Rs power calculator (https://eda.nc3rs.org.uk/experimental-design-group) was used using data from preliminary data (*n* = 3 or 4) to calculate sample size based on significance effect of 0.05, power 0.8 and on a estimated size effect of: Fig. 2g: 50% Fig. 3d: 40% Fig. 4e: 15% Fig. 5a: 50% for CNP (mice are haplo-insufficient) and 20% for others.

### Reporting summary

Further information on research design is available in the Nature Portfolio Reporting Summary linked to this article.

## Online content

Any methods, additional references, Nature Portfolio reporting summaries, source data, extended data, supplementary information, acknowledgements, peer review information; details of author contributions and competing interests; and statements of data and code availability are available at https://doi.org/10.1038/s41593-026-02383-0.

## Supplementary information


Supplementary InformationSupplementary Tables 1a,b and 2 and Supplementary Methods.
Reporting Summary
Supplementary Video 1Individual *z*-steps of the cell shown in Fig. 1l.
Supplementary Video 23D view of the cell in Fig. 1l.
Supplementary Video 3Myelin peroxisome in internodal myelin; related to Fig. 2.
Supplementary Video 4Myelin peroxisome entering paranodal loop; related to Fig. 2.
Supplementary Video 5Myelin peroxisome traversing multiple paranodal loops; related to Fig. 2.
Supplementary Video 6Zoom of myelin peroxisome traversing paranodal loops; related to Fig. 2.
Supplementary Video 7βTubulin IV in a tdTomato-labeled myelinating oligodendrocyte; related to Fig. 2.
Supplementary Video 8Oligodendrocyte peroxisomes in the soma and processes; related to Fig. 3.
Supplementary Video 9Timelapse of oligodendrocyte peroxisome movement on inert nanofibers; related to Extended Data Fig. 4.
Supplementary Video 10Timelapse of oligodendrocyte peroxisomes movement in vivo; related to Fig. 3.


## Source data


Source DataSource data for Figs. 2–6 and Extended Data Fig. 5.


## Data Availability

Raw data will be made available upon reasonable request. Source data are provided with this paper.

## Code availability

No custom software codes were used in the data analysis for this paper.
