# Supplementary material for: An activity-modulated transport route across myelin (TRAM) for motor-driven organelle transfer in oligodendrocytes
Source: Nat Neurosci. 2026 Jul 31;29(9):2110–23. doi: 10.1038/s41593-026-02383-0 (PMC13533835; doi:10.1038/s41593-026-02383-0)
Supplement: Supplementary file 1 [file 41593_2026_2383_MOESM1_ESM.pdf]

nature neuroscience

Supplementary information

https://doi.org/10.1038/s41593-026-02383-0

An activity-modulated transport route across myelin (TRAM) for motor-driven organelle transfer in oligodendrocytes

In the format provided by the authors and unedited

Supplementary Information

Supplementary Table 1A. Motor proteins identified by label-free mass spectrometry in myelin.

Supplementary Table 1B. Selected proteins identified by label-free mass spectrometry in myelin.

Supplementary Table 2. *Mbp* probes used for smFISH

Supplementary Methods. Overview images of two optic nerve cross sections on a TEM grid.

Supplementary Table 1A. Motor proteins identified by label-free mass spectrometry in myelin purified from the brains of c57Bl/6N mice (MM) according to Jahn et al. (2020) or in myelin purified from the white matter of humans (HS) according to Gargareta et al. (2022).

Motor protein	Akronym	MM	HS	Function
Dynein 1 heavy chain	DYNC1H1	1	1	Motor protein
Dynein 1 intermediate chain 1	DYNC1I1	0	1	Motor protein
Dynein 1 intermediate chain 2	DYNC1I2	1	1	Motor protein
Dynein 1 light intermediate chain 1	DYNC1LI1	1	1	Motor protein
Dynein 1 light intermediate chain 2	DYNC1LI2	1	1	Motor protein
Dynein light chain 1	DYNLL1	1	1	Motor protein
Dynein light chain 2	DYNLL2	1	1	Motor protein
Kinesin heavy chain 5A	KIF5A	0	1	Motor protein
Kinesin heavy chain 5B	KIF5B	1	1	Motor protein
Kinesin heavy chain 5C	KIF5C	1	1	Motor protein
Kinesin light chain 1	KLC1	1	1	Motor protein
Kinesin light chain 2	KLC2	0	1	Motor protein
Kinesin-like protein 2A	KIF2A	0	1	Motor protein
Kinesin-like protein 21A	KIF21A	0	1	Motor protein
Myosin 1d	MYO1D	1	1	Motor protein
Myosin 1e	MYO1E	1	0	Motor protein
Myosin 5a	MYO5A	1	1	Motor protein
Myosin 5d	MYO5D	0	1	Motor protein
Myosin 6	MYO6	1	0	Motor protein
Myosin 18a	MYO18A	1	1	Motor protein
Myosin heavy chain 9	MYH9	1	1	Motor protein
Myosin heavy chain 10	MYH10	1	1	Motor protein
Myosin heavy chain 11	MYH11	0	1	Motor protein
Myosin heavy chain 14	MYH14	1	1	Motor protein
Myosin light chain 6	MYL6	1	1	Motor protein
Myosin light chain 6b	MYL6B	0	1	Motor protein
Myosin light chain 12b	MYL12B	1	0	Motor protein

Supplementary Table 1B. Selected proteins identified by label-free mass spectrometry in myelin purified from the brains of c57Bl/6N mice (MM) according to Jahn et al. (2020) or in myelin purified from the white matter of humans (HS) according to Gargareta et al. (2022).

Tubulin/actin associated proteins	Akronym	MM	HS	Function
Centractin alpha	ACTR1A	1	1	Binds microtubules
Centractin beta	ACTR1B	1	1	Binds microtubules
Cofilin 1	CFL1	1	1	Actin modulating
Cofilin 2	CFL2	1	1	Actin modulating
Cyclic nucleotide phosphodiesterase	CNP	1	1	Binds tubulins
Dynactin subunit 1	DCTN1	1	1	Binds microtubules
Dynactin subunit 2	DCTN2	1	1	Binds microtubules
Dynactin subunit 3	DCTN3	0	1	Binds microtubules
Dynactin subunit 4	DCTN4	1	1	Binds microtubules
Histone deacetylase 11	HDAC11	1	1	Tubulin-modifying
Microtubule-associated protein 1A	MAP1A	1	1	Tubulin-modifying
Microtubule-associated protein 1B	MAP1B	1	1	Tubulin-modifying
Microtubule-associated protein 2	MAP2	1	0	Tubulin-modifying
Microtubule-associated protein 4	MAP4	1	1	Tubulin-modifying
Microtubule-associated protein 6	MAP6	1	1	Tubulin-modifying
Sirtuin 2	SIRT2	1	1	Tubulin-modifying
Tubulin alpha	TUBA	1	1	Tubulin
Tubulin beta	TUBB	1	1	Tubulin
Tubulin polymerization-promoting protein	TPPP	1	1	Tubulin-modifying
Tubulin polymerization-promoting protein 3	TPPP3	1	1	Tubulin-modifying

Supplementary Table 2. *Mbp* probes used for smFISH

Mbp probe sequences

mMbp-exon_01	gcctgtctttgaaggtgt	mMbp-exon_23	cggctccacgggattaag
mMbp-exon_02	gaagctcgtcggactctg	mMbp-exon_24	gttttaaccagtcggggt
mMbp-exon_03	tggggtcttcttggatgg	mMbp-exon_25	gtcagggctgagaagac
mMbp-exon_04	gtgagggtctcttctgtg	mMbp-exon_26	acctccggagtcgaacaa
mMbp-exon_05	tggccaggtacttggatc	mMbp-exon_27	cttggaagggtgtccgtg
mMbp-exon_06	gcatggtccatggtactt	mMbp-exon_28	acggaagctggaggggg
mMbp-exon_07	ctgtgccttgggaggaag	mMbp-exon_29	ccaagactgtctgatcct
mMbp-exon_08	aagaagcgcccgatggg	mMbp-exon_30	aagcccccgttgtataag
mMbp-exon_09	ctgctttagccagggtac	mMbp-exon_31	ccacgctcgaaatcagct
mMbp-exon_10	gcatgagagggcagagg	mMbp-exon_32	cagcgtgttctcctaagt
mMbp-exon_11	tgtacatgtggcacagcc	mMbp-exon_33	atttggcggccacacata
mMbp-exon_12	cagggagccataatgggt	mMbp-exon_34	ggagccactgaggagaa
mMbp-exon_13	gttttcatcttgggtccg	mMbp-exon_35	cctgggctctgagaggaa
mMbp-exon_14	gaggtggtgttcgaggtg	mMbp-exon_36	caccatgagaagtggcca
mMbp-exon_15	atctgctgagggacaggc	mMbp-exon_37	tgtaccaatgggcactgc
mMbp-exon_16	tagccaaatcctggcttc	mMbp-exon_38	ctttctaggaggcaggga
mMbp-exon_17	atagtcggaagctctgcc	mMbp-exon_39	cccccctaaagctaagaa
mMbp-exon_18	ttgaatcccttgtgagcc	mMbp-exon_40	gcagcgactcgattcagt
mMbp-exon_19	tttggaaagcgtgccctg	mMbp-exon_41	gtgtggggctctttggaa
mMbp-exon_20	atgggagatccagagcg	mMbp-exon_42	acatcaaccatcacctgc
mMbp-exon_21	gagggcaggattcggga	mMbp-exon_43	acccacactctactcaga
mMbp-exon_22	gggtctgctctaactagc	mMbp-exon_44	accctcacgttattgtgg
		mMbp-exon_45	aaacactcccgtgggaca
		mMbp-exon_46	ggtcgttcagtcacactg
		mMbp-exon_47	gtctggacgaagccatgg
		mMbp-exon_48	tagtaggtgcttctgtcc

Supplementary Methods

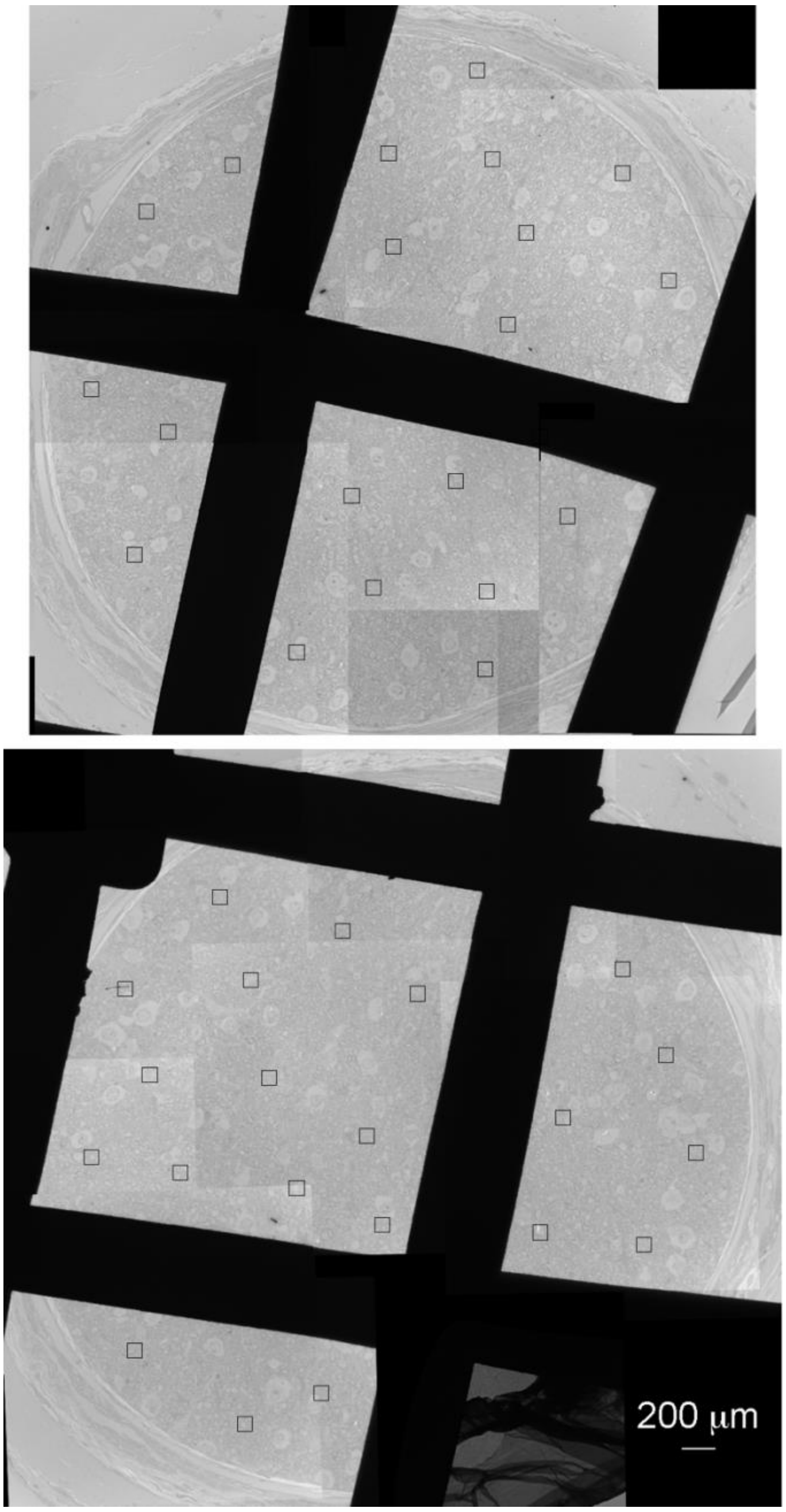

Overview images of two optic nerve cross sections on a TEM grid. 'Stitched' images of two ~50 nm thick sections of the same nerve on a TEM grid, to illustrate how sampling was performed and influenced by grid bars or resin shrinkage. TEM images (represented by small squares, drawn approximately to scale) were captured at random across the nerve section by a blinded experimenter. The precise point of image capture was influenced by the location of the grid bars or resin shrinkage (bottom right corner of lower image). Images were captured from only one section per nerve; two sections are shown here to illustrate how the location of the grid bars on the nerve influence image capture.
