# Supplementary material for: An activity-modulated transport route across myelin (TRAM) for motor-driven organelle transfer in oligodendrocytes
Source: Nat Neurosci. 2026 Jul 31;29(9):2110–23. doi: 10.1038/s41593-026-02383-0 (PMC13533835; doi:10.1038/s41593-026-02383-0)
Supplement: Supplementary file 2 [file 41593_2026_2383_MOESM2_ESM.pdf]

nature portfolio

Corresponding author(s): Julia Edgar

Last updated by author(s): 24-Apr-2026

# Reporting Summary

Nature Portfolio wishes to improve the reproducibility of the work that we publish. This form provides structure for consistency and transparency in reporting. For further information on Nature Portfolio policies, see our Editorial Policies and the Editorial Policy Checklist.

## Statistics

For all statistical analyses, confirm that the following items are present in the figure legend, table legend, main text, or Methods section.

| n/a | Confirmed | |
|---|---|---|
| ☐ | ☒ | The exact sample size (*n*) for each experimental group/condition, given as a discrete number and unit of measurement |
| ☐ | ☒ | A statement on whether measurements were taken from distinct samples or whether the same sample was measured repeatedly |
| ☐ | ☒ | The statistical test(s) used AND whether they are one- or two-sided<br>*Only common tests should be described solely by name; describe more complex techniques in the Methods section.* |
| ☒ | ☐ | A description of all covariates tested |
| ☐ | ☒ | A description of any assumptions or corrections, such as tests of normality and adjustment for multiple comparisons |
| ☐ | ☒ | A full description of the statistical parameters including central tendency (e.g. means) or other basic estimates (e.g. regression coefficient) AND variation (e.g. standard deviation) or associated estimates of uncertainty (e.g. confidence intervals) |
| ☐ | ☒ | For null hypothesis testing, the test statistic (e.g. $F$, $t$, $r$) with confidence intervals, effect sizes, degrees of freedom and $P$ value noted<br>*Give P values as exact values whenever suitable.* |
| ☒ | ☐ | For Bayesian analysis, information on the choice of priors and Markov chain Monte Carlo settings |
| ☒ | ☐ | For hierarchical and complex designs, identification of the appropriate level for tests and full reporting of outcomes |
| ☒ | ☐ | Estimates of effect sizes (e.g. Cohen's *d*, Pearson's *r*), indicating how they were calculated |

*Our web collection on statistics for biologists contains articles on many of the points above.*

## Software and code

Policy information about availability of computer code

Data collection

In vivo imaging: Images captured using Zeiss LSM 7MP microscope equipped with a BiG GaAsP detector using a modellocked Ti:sapphire laser (Coherent Ultra). Image stacks were acquired using a Zeiss W plan-apochromat x20/1.0 NM water immersion objective.
In vitro live imaging: Timelapse images were acquired using an inverted Zeiss Observer Z1 Spinning Disc confocal microscope, equipped with a Yokogawa CSU X1 filter wheel and spinning disc unit, a Photometrics Evolve 512 delta TEM CCD camera and a 63x/1.4 oil Pln Apo objective, using Zen software (2012, Blue Edition).
Immunoelectron microscopy: Sections were analysed on a LEO EM912 Omega (Zeiss) and micrographs obtained with an on axis 2048x2048 CCD camera (TRS)
White matter morphometry: Images were captured on a JEOL 120 EX transmission electron microscope or a TEM912AB electron microscope (aged mice). Olympus I TEM Software or ImageSP software (TRS, SysProg, ImageSP, vers1.1.4.62) (for aged mice) was used for image acquisition.
smFISH: Section were imaged using an Olympus SpinSR (CSU-W1) spinning disk confocal microscope with a Prime BSI sCMOS camera.
Immunohistochemistry, immunofluorescence: Images were taken using ZEN 2012 blue edition software, except cells on nanofibres, which were imaged using Image-Pro Plus 6 software (Media Cybernetics, Silver Spring, MD).
Western blots: Immunoblots were scanned using the Intas ChemiCam system (Goettingen) or using the Li-Cor C-DiGit blot scanner (Glasgow).
Electrophysiology: Optic nerve recordings were acquired using an Axon Digidata 1550B using Clampex 11.3 (Molecular Devices Ltd, Wokingham, Berkshire, UK). Neuronal recordings were acquired using Axopatch 200B amplifier with a National Instruments NIDAQ-MX digital acquisition system.
Seahorse OCR and ECAR measurements following treatment of neuronal cell cultures with neuromodulators were acquired using Agilent Seahorse Wave Desktop software.

| | |
|---|---|
| Data analysis | In vivo peroxisome motility: Timelapses were analysed in ImageJ using TurboReg plugin. Image stacks were analysed inIMageJ using Poorman3Dreg plugin and Correct3D drift plugin.<br>Electrophysiology - Action potential firing data was analysed with WinEDR V3.9.1 software (Strathclyde electrophysiology software, http://spider.science.strath.ac.uk/sipbs/software_ses.htm)<br>In vitro peroxisome motility - Peroxisome motility was tracked manually using MTrackJ plugin in ImageJ2 (Version: 2.14.0/1.54f). Numbers of peroxisomes in oligodendrocyte processes or sheaths were counted manually using the multi-point tool in Fiji (Schindelin et al., 2012).<br>Immunofluoresence - ImageJ2 (Version: 2.14.0/1.54f) was used to quantify structures or count cells or organelles (e.g. peroxisomes).<br>Immunohistochemistry - Images were color deconvoluted using the "Color Deconvolution" (1.3) plugin in ImageJ and APP spheroids were quantified manually using the "Cell counter" plugin from ImageJ.<br>White matter morphology : ImageJ2 (Version: 2.14.0/1.54f) was used to quantify structures using the Image J Cell Counter plug-in.<br>Western Blots - signal intensity of protein band was measured in ImageJ2 (Version: 2.14.0/1.54f)<br>Seahorse neuromodulator data was analysed using Seahorse XF HS Mini Bioanalyser (Agilent) |

For manuscripts utilizing custom algorithms or software that are central to the research but not yet described in published literature, software must be made available to editors and reviewers. We strongly encourage code deposition in a community repository (e.g. GitHub). See the Nature Portfolio guidelines for submitting code & software for further information.

## Data

Policy information about availability of data

All manuscripts must include a data availability statement. This statement should provide the following information, where applicable:

- Accession codes, unique identifiers, or web links for publicly available datasets
- A description of any restrictions on data availability
- For clinical datasets or third party data, please ensure that the statement adheres to our policy

Raw data will be made available upon a reasonable request.

## Research involving human participants, their data, or biological material

Policy information about studies with human participants or human data. See also policy information about sex, gender (identity/presentation), and sexual orientation and race, ethnicity and racism.

| | |
|---|---|
| Reporting on sex and gender | NA |
| Reporting on race, ethnicity, or other socially relevant groupings | NA |
| Population characteristics | NA |
| Recruitment | NA |
| Ethics oversight | NA |

Note that full information on the approval of the study protocol must also be provided in the manuscript.

# Field-specific reporting

Please select the one below that is the best fit for your research. If you are not sure, read the appropriate sections before making your selection.

☒ Life sciences ☐ Behavioural & social sciences ☐ Ecological, evolutionary & environmental sciences

For a reference copy of the document with all sections, see nature.com/documents/nr-reporting-summary-flat.pdf

# Life sciences study design

All studies must disclose on these points even when the disclosure is negative.

| | |
|---|---|
| Sample size | Sample size was between 3-9 independent experiments (i.e. 3-9 controls and 3-9 matched experimental samples), except in two cases, where only two matched pairs were analysed (Figure 5A [anti-MOG on 1 year samples] and Figure 5D ( inner tongue organelles at P40 and P120). No statistical tests were undertaken on these.<br>The NC3Rs power calculator (https://eda.nc3rs.org.uk/experimental-design-group) was used using data from preliminary data (n = 3 or 4) to calculate sample size based on significance effect of 0.05, power 0.8 and on a estimated size effect of:<br>Figure 2G: 50%<br>Figure 3D: 40%<br>Figure 4E: 15%<br>Figure 5A: 50% for CNP (mice are haplo-insufficient) and 20% for others. |

| | |
|---|---|
| Data exclusions | Peroxisome motility: Data were excluded if there was <5 myelin peroxisomes observable in a sheath, to minimise skewing of percentage motility data. This is stated in our M&M and was applied across all experimentals conditions. Sheaths were excluded if peroxisomes move out of the focal plane due to drift. Peroxisomes were excluded from analysis if the total distance covered in 10 minutes was less than 3 um.<br>Cell cultures were excluded from analysis if they did not meet quality control criteria i.e. that tdTomato-rendered myelin did not appear smooth, but instead contained 'blebbing'.<br>For morphometry, samples were (partially) excluded if the fixation was not adequate for identifying specific features like organelles. One cKif21b knockout optic nerve was excluded from morphometry because it appeared shrunken and abnormal; another due to an ID issue. |
| Replication | To demonstrate reproducibility all experiments were repeated a minimum of two times but the majority were independently repeated at least three times. In experiments using myelinating cultures, 'independent experiment' refers to independent cell cultures prepared from different pregnant 'reporter' mice. The nanofibre experiment (supplementary Figure 4) was performed on one cell culture. The accompanying oligodendrocyte on glass experiment (supplementary Figure 4) was performed using two independent cell cultures.<br>For electron microscopy studies and optic nerve electrophysiology, each n represents one animal (one nerve per animal for TEM; both nerves used for electrophysiology, one stimulated one not). For in vivo peroxisome quantification, 4 independent animals were examined. |
| Randomization | Peroxisome quantification in vitro samples: Treatments were applied randomly to 'sister' cultures, meaning that for each independent culture (pooled embryos from one pregnant mouse), individual Petri-dishes, each containing approx. 450,000 cells, were randomly assigned to treatments (nocodazole versus vehicle; TTX or PTX versus vehicle).<br>KIF21b conditional KO and controls for TEM and western blotting: mice were bred to produce littermates of the required genotypes. Litter mates (usually) or closely related relatives from an inbred colony were compared at each timepoint based on availability of the appropriate genotype at the required age.<br>For optic nerve electrophysiology, nerves were paired; from the same animal, one was stimulated and one not. |
| Blinding | Image analysis: For morphometry of TEM images, animals were known to the experimenter by ID number only. The ID provided no clue to the animal's genotype.<br>Peroxisome motility (in vitro and in vivo) - The experimenter recorded the time-lapse images, knowing the treatment condition. However, the time lapse image files were blinded (renamed by an independent experimenter) before the experimenter performed the quantification.<br>Blinding was not performed for western blots or cell electrophysiology but in both cases, data were generated using equipment and software that performs non-subjective readouts. |

# Reporting for specific materials, systems and methods

We require information from authors about some types of materials, experimental systems and methods used in many studies. Here, indicate whether each material, system or method listed is relevant to your study. If you are not sure if a list item applies to your research, read the appropriate section before selecting a response.

## Materials & experimental systems

| n/a | Involved in the study | |
|---|---|---|
| ☐ | ☒ | Antibodies |
| ☒ | ☐ | Eukaryotic cell lines |
| ☒ | ☐ | Palaeontology and archaeology |
| ☐ | ☒ | Animals and other organisms |
| ☒ | ☐ | Clinical data |
| ☒ | ☐ | Dual use research of concern |
| ☒ | ☐ | Plants |

## Methods

| n/a | Involved in the study | |
|---|---|---|
| ☒ | ☐ | ChIP-seq |
| ☒ | ☐ | Flow cytometry |
| ☒ | ☐ | MRI-based neuroimaging |

## Antibodies

| | |
|---|---|
| Antibodies used | anti-Kif21b, Sigma-Aldrich, HPA027274, Polyclonal<br>anti-β-Actin, Sigma-Aldrich, A1978, Clone AC-15<br>anti-β tubulin III, Merck, T8578, Clone 2G10<br>anti-β tubulin IV, Abcam, ab11315, Clone ONS.1A6<br>anti-MBP, Abcam, ab7349, Clone 12<br>anti-CNP, Abcam, ab6319, Clone 11-5B<br>anti-Ermin, Merck, MABN323, Clone 160<br>anti-tyrosinated tubulin, Merck, T9028, Clone TUB-1A2<br>anti-acetylated tubulin, Merck, T7451, Clone 6-11B-1<br>anti-detyrosinated tubulin, Abcam, ab48389, Polyclonal<br>anti-MOG, gifted by Professor Christopher Linington, Clone Z2.<br>anti-Caspr1, provided by Professor E Peles<br>anti-MCT1, provided by Katherine Kusch, rabbit polyclonal (Stumpf et al., 2019). "For generation of MCT1 (SLC16a1) antisera, rabbits were immunized with the intracellular peptide 221–236 of mouse MCT1 (CDANTDLIGGSPKGEKL). Anti-MCT1 antibodies were purified by affinity chromatography."<br>anti-MAC3 Pharmingen 553322, rat<br>anti-APP Millipore MAB348/3200582, mouse |

anti-Iba1 Abcam ab5076, rat
anti ATP1A1 Abcam ab7671, mouse
anti-PEX14 Abcam ab183885, rabbit
anti-PMP70 Abcam ab3421, rabbit
anti-Catalase Rockland 200-4151, Rabbit

Validation

Anti-KIF21b was preadsorbed against KIF21B knockout mouse brain and validated in western blotting using KIF21b knockout mouse brain (Supplemenatry Figure 6) and Source Data 'Antibody optimisation WB June 2018'
Anti-CNP was previously validated by us on Cnp1 knockout mouse CNS tissue.
Anti-MOG has been demonstrated by us to bind to the surface of myelin in murine myelinating cell cultures.
Other antibodies used for western blotting were considered valid based on the molecular weight of the band they recognised.
Antibodies used for immunostaining were considered valid based on the staining pattern they produced e.g. anti-beta tubulin IV lablled myelinating oligodendrocytes, in vitro, whereas anti-beta tubulin III labelled neuronal cells in vitro; anti-Caspr1 labelled paranodal regions of myelin sheaths in vitro and in vivo. For immunocytochemistry or immunohistochemistry, each antibody was run in parallel with a no primary control.

## Animals and other research organisms

Policy information about studies involving animals; ARRIVE guidelines recommended for reporting animal research, and Sex and Gender in Research

Laboratory animals

Transgenic mice had been crossed onto a C57BL/6 Charles River background, as described in the original publications and/or following acquisition by us harbouring combinations of transgenes encoding tamoxifen-inducible PLPCreERT2 {Leone, 2003 7898 /id; Weber, 2001 7163 /id}, the "stop-floxed" Cre reporter gene ß-actin-tdTomato {Madisen, 2010 10467 /id} and Cnp-mEos2-PTS1 {Richert, 2014 10701 /id}.
Conditional Kif21b knockout transgenic mice and 'reporter' mice harbouring for various combinations of tamoxifen-inducible PLPCreERT2.
cKif21b KO mice (Muhia et al., 2016): By intercrossing Kif21blacZ/neo mutants with mice expressing FLIP recombinase (129S4/SvJaeSor-Gt(ROSA)26Sortm1(FLP1)Dym/J; backcrossed into C57BL/6N for at least 10 generations) (Farley et al., 2000) the lacZ/neo cassette was excised in vivo, yielding the "conditional ready" heterozygous flox allele in mice carrying the Kif21f KIF21btm2a(KOMP)Wtsi allele (also termed Kif21bflox/+).
Tubba4 mice - Highly chimeric animals were crossed to C57Bl/6N females yielding Tubb4atm1a(EUCOMM)Hmgu mutant mice (also termed Tubb4alacZ/neo). By intercrossing Tubb4alacZ/neo mutants with mice expressing FLIP recombinase (129S4/SvJaeSor-Gt(ROSA)26Sortm1(FLP1)Dym/J; backcrossed into C57BL/6N for at least 10 generations).
CD-1 mice were used for the optic nerve electrophysiology.
C57BL/6J mice were aged between P40 and 24 months and CD-1 mice were 30-45 days old.

Wild animals

The study did not involve wild animals

Reporting on sex

Both male and female cKif21b KO and control mice were used for electron microscopy and western blotting. Mice were grouped according to age and litter e.g. one group of P60 animals encompassed each of three genotypes, usually from the same litter, or occasionally, from closely related relatives from the same in-bred colony. Due to this, there are not sufficient numbers to disaggregate data based on sex. For the aging study (Figure 6) all mice were males. Myelinating cell cultures were each generated by pooling all embryos from a pregnant mouse; therefore both sexes were included. CD-1 mice were all male. Cnp null mice & controls were male (Figure 5 C) or mixed sex (Figure 5D). Beta-tubulin 4 ko mice and controls were male. When mixed sex animals were used, they were ascribed to a condition (control or experimental) according to genotype, so there was no predetermined bias is the distribution of the sexes.

Field-collected samples

Study did not involve samples collected from the field

Ethics oversight

Animal experiment carried out at the University of Glasgow were approved by the University of Glasgow Animal Welfare and Ethical Review Board (AWERB), licenced under the UK Animals (Scientific Procedures) Act of 1986, and conducted according to the ARRIVE guidelines.
Animal experiments arried out at the Max Planck Institute for Multidisciplinary Sciences (MPI-NAT) were performed in accordance with the German animal protection law (TierSchG) and approved by the Niedersächsisches Landesamt für Verbraucherschutz und Lebensmittelsicherheit (LAVES) under license 33.19-42502-04-17/2409, if subject to approval. Procedures were supervised by the animal welfare officer for the MPI-NAT, Göttingen, Germany. The animal facility is registered at the MPI-NAT is registered according to §11 Abs. 1 TierSchG.
For electrophysiology of optic nerves, experiments were approved by the University of Nottingham Animal Care and Ethics Committee and carried out in accordance with the Animals (Scientific Procedures) Act 1986 under appropriate authority of establishment, project and personal licences.
For the in vivo peroxisome imaging, animal experiments were conducted in accordance with protocols approved by the Animal Care and Use Committee at the University of Colorado Anschutz Medical Campus.

Note that full information on the approval of the study protocol must also be provided in the manuscript.

## Plants

| | |
|---|---|
| Seed stocks | NA |
| Novel plant genotypes | NA |
| Authentication | NA |
