# Supplementary material for: An activity-modulated transport route across myelin (TRAM) for motor-driven organelle transfer in oligodendrocytes
Source: Nat Neurosci. 2026 Jul 31;29(9):2110–23. doi: 10.1038/s41593-026-02383-0 (PMC13533835; doi:10.1038/s41593-026-02383-0)
Supplement: Supplementary file 3 [file 41593_2026_2383_MOESM13_ESM.zip › Zip file for NN with source data/Table of Contents Source Data files.docx]

1. Antibody optimisation WB June 2018
2. ax degen_density WT 6_24m _SGnew
3. Figure 4 uncropped blots P60 ON for NN
4. Figure 4E Morphometry cKif21b ko optic nerve retinal end
5. Figure 5A uncropped blots spinal cord myelin
6. Figure 5A western blot raw data
7. Figure 5C uncropped blots MCT1 in Cnp1 KO
8. Figure 6 uncropped blots of MCT1 in aged animals
9. IT Area nach axon diameter aufgeschlüsselt WT 6_24m
10. Perox motility in vivo Figure 3
11. Perox motilty by sheath Figures 2 & 3
12. Quantification_IT_2019 Ulrike
13. Supp Fig 5 source data ON axon densities summary
14. Supp Fig 5 source data ON G-ratio summary
15. Table of Contents Source Data files
